# Characterisation of detergent-insoluble membranes in pollen tubes of *Nicotiana tabacum* (L.)

**DOI:** 10.1242/bio.201410249

**Published:** 2015-02-20

**Authors:** Alessandra Moscatelli, Assunta Gagliardi, Lilly Maneta-Peyret, Luca Bini, Nadia Stroppa, Elisabetta Onelli, Claudia Landi, Monica Scali, Aurora Irene Idilli, Patrick Moreau

**Affiliations:** 1 Dipartimento di Bioscienze, Università degli Studi di Milano, Via Celoria 26, 20133 Milan, Italy; 2 Laboratorio di Proteomica Funzionale, Dipartimento di Scienze della Vita, Università degli Studi di Siena, Via Aldo Moro 2, 53100 Siena, Italy; 3 Laboratoire de Biogenèse Membranaire, Université Bordeaux Segalen, 71 Avenue Edouard Bourlaux, 33883 Villenave d'Ornon, France; 4 Dipartimento di Scienze della Vita, Università degli Studi di Siena, Via P. A. Mattioli 4, 53100 Siena, Italy; * Present address: Institute of Biophysics, National Research Council and FBK, 38123 Trento, Italy.

**Keywords:** *Nicotiana tabacum* (L.), Pollen tube, Membrane microdomains

## Abstract

Pollen tubes are the vehicle for sperm cell delivery to the embryo sac during fertilisation of Angiosperms. They provide an intriguing model for unravelling mechanisms of growing to extremes. The asymmetric distribution of lipids and proteins in the pollen tube plasma membrane modulates ion fluxes and actin dynamics and is maintained by a delicate equilibrium between exocytosis and endocytosis. The structural constraints regulating polarised secretion and asymmetric protein distribution on the plasma membrane are mostly unknown. To address this problem, we investigated whether ordered membrane microdomains, namely membrane rafts, might contribute to sperm cell delivery. Detergent insoluble membranes, rich in sterols and sphingolipids, were isolated from tobacco pollen tubes. MALDI TOF/MS analysis revealed that actin, prohibitins and proteins involved in methylation reactions and in phosphoinositide pattern regulation are specifically present in pollen tube detergent insoluble membranes. Tubulins, voltage-dependent anion channels and proteins involved in membrane trafficking and signalling were also present. This paper reports the first evidence of membrane rafts in Angiosperm pollen tubes, opening new perspectives on the coordination of signal transduction, cytoskeleton dynamics and polarised secretion.

## INTRODUCTION

The mosaic fluid model of Singer and Nicolson (Singer and Nicolson, 1972) was recently reviewed in light of a new concept, representing cell membranes as a mosaic of highly structured microdomains, called membrane rafts, alternating with less organised regions (Lingwood and Simons, 2010). *In vitro* studies showed that lipids with high melting temperature spontaneously partition into detergent insoluble microdomains (DIMs) (Ahmed et al., 1997; Schroeder et al., 1998; Brown and London, 2000; London and Brown, 2000). The prerequisite for phase separation and detergent insolubility resides in the close lateral associations established between sterols, sphingolipids and highly saturated phospholipids (Ahmed et al., 1997; Brown and London, 2000; Dietrich et al., 2001; London, 2002; Silvius, 2003). In particular, the association between rigid sterol molecules and sphingolipids leads to a more organised, liquid-ordered phase (L_o_) (Ohvo-Rekilä et al., 2002; Silvius, 2003) that coexists in the same membrane with liquid-disordered (L_d_) domains (Brown and London, 1997; Edidin, 2003).

The membrane raft concept led to reconsideration of the structural organisation of the plasma membrane (PM) and mechanisms controlling membrane trafficking in eukaryotes. Although lipids themselves are a prerequisite for phase partition, specific protein recruitment also helps these domains to compartmentalise cell processes in the PM (Schroeder et al., 1994; Brown and London, 1997) and to regulate protein sorting to different cell destinations (Muñiz and Zurzolo, 2014). Early evidence showed that sorting of sterol-sphingolipid enriched domains directed to the apical PM occurs in the trans-Golgi network (TGN) (Simons and van Meer, 1988; Simons and Ikonen, 1997; Surma et al., 2012), while a sterol-sphingolipid-independent mechanism was suggested for the basolateral secretory pathway in polarised epithelial cells (Mays et al., 1995; Keller and Simons, 1998). Recent data suggested a more complex scenario, in which oligomerisation of GPI-anchored proteins on Golgi membranes leads small raft domains to coalesce into more stable platforms that in turn contribute to membrane curvature and vesicle fission during apical sorting (Paladino et al., 2004; Paladino et al., 2007). Lipid raft domains are also involved in endocytosis (Kirkham and Parton, 2005; Eyster et al., 2009); studies of GPI-anchored proteins showed internalisation pathways based on caveolin-coated domains (Anderson, 1998) or on the integrity of DIMs (Sabharanjak et al., 2002). Membrane microdomains also take part in cell processes, such as signal transduction (Simons and Toomre, 2000) and cytoskeleton organisation (Falk et al., 2004; Chichili and Rodgers, 2009) which further promote polarised morphogenesis in animals and fungi (Cheng et al., 2001; Bagnat and Simons, 2002; Martin and Konopka, 2004).

The asymmetric distribution of organelles, proteins and lipids on the PM in pollen tubes is a distinctive feature and the fundamental requirement for tip growth and sexual reproduction in higher plants (Moscatelli and Idilli, 2009). Vesicles accumulate in the tip or clear zone of pollen tubes, while larger organelles are retained in the shank (Hepler et al., 2001; Cheung and Wu, 2008). Recent data has shown that distinct actin filament- and microtubule-dependent secretory pathways are involved in directing membranes to the apical flanks/shank and to the central area of the apex, respectively (Kroeger et al., 2009; Moscatelli et al., 2012; Idilli et al., 2013). In addition, PM internalisation and membrane recycling in the apex suggest that the clear zone could be the major area of membrane sorting in pollen tubes (Idilli et al., 2013). Although RabGTPases, such as NtRab2 and NtRab11 (Cheung et al., 2002; de Graaf et al., 2005), have been identified as regulators of membrane trafficking, the identification of additional players modulating membrane sorting to the apex rather than the shank calls for further investigation.

Targeted secretion and fine regulation of endocytosis are responsible for the asymmetrical distribution of proteins and lipids along the pollen tube PM (Moscatelli and Idilli, 2009). Studies on *Arabidopsis* and tobacco pollen tubes also showed that Rac-RopGTPase is localised in a restricted apical PM domain where it works as a general switch to control Ca^2+^ release, AF dynamics and polarised exocytosis (Kost et al., 1999; Fu et al., 2001; Gu et al., 2003; Lee et al., 2008; Zonia, 2010). In addition, the effector of Rac-RopGTPase is a phosphatidylinositol monophosphate kinase, responsible for phosphatidyl inositol 4,5-bisphosphate (PIP_2_) accumulation in the apex (Hartwig et al., 1995; Kost et al., 1999). The high content of PIP_2_ in a restricted area of the apex is in turn maintained by phospholipase C, localised in the apical flanks and involved in PIP_2_ hydrolysis (Yang and Kazanietz, 2003; Helling et al., 2006). Oscillations in Ca^2+^ concentrations at the very tip and the H^+^ATPase-dependent alkaline band behind the tip (Feijó et al., 1999; Certal et al., 2008) regulate AFs (Cárdenas et al., 2008) and fast membrane flows to the apical flanks of the PM during pulsed growth (Zonia and Munnik, 2008; Bove et al., 2008; Moscatelli et al., 2012). The high PIP_2_ content in the apex was also thought to facilitate the fusion of SVs (Kost et al., 1999) and to be a site of clathrin-dependent endocytosis (Zhao et al., 2010). In *Arabidopsis*, sterol accumulation also marked the site of root hair emergence, while polarised distribution of sterols on tip-localised SVs and apical PM was required for root hair elongation (Ovecka et al., 2010). Since it has been shown that the polarised distribution of sterols has a crucial effect on cell polarisation and that half the PIP_2_ occurs in DIMs of tobacco cells (Furt et al., 2010), we hypothesised that membrane rafts could be the structural determinants of cell asymmetry during pollen tube growth. To address this problem, experiments were performed to identify DIMs in Angiosperm pollen tubes.

DIMs were purified from tobacco pollen tube microsomes using increasing detergent/protein ratios. Lipid analysis showed that DIMs were characterised by high sterol content, increased content of phospholipids with saturated fatty acids and increased content of hydroxylated very long chain fatty acid (VLCFA) sphingolipids. Protein identification by MALDI TOF/MS revealed that pollen tube DIMs specifically displayed actin, prohibitins and proteins regulating phosphoinositide composition and methylation reactions. They also showed proteins that have been identified in DIMs isolated from plant somatic cell PM, such as voltage dependent anion channels (VDACs), tubulins and proteins involved in membrane trafficking and signalling. This data, together with the effects of sterol deprivation on DIMs and on cell morphology, suggested that membrane microdomains play a role in the asymmetric growth pattern of Angiosperm pollen tubes.

## RESULTS

### Detergent-insoluble membranes from tobacco pollen tubes

**Fig. 1.**
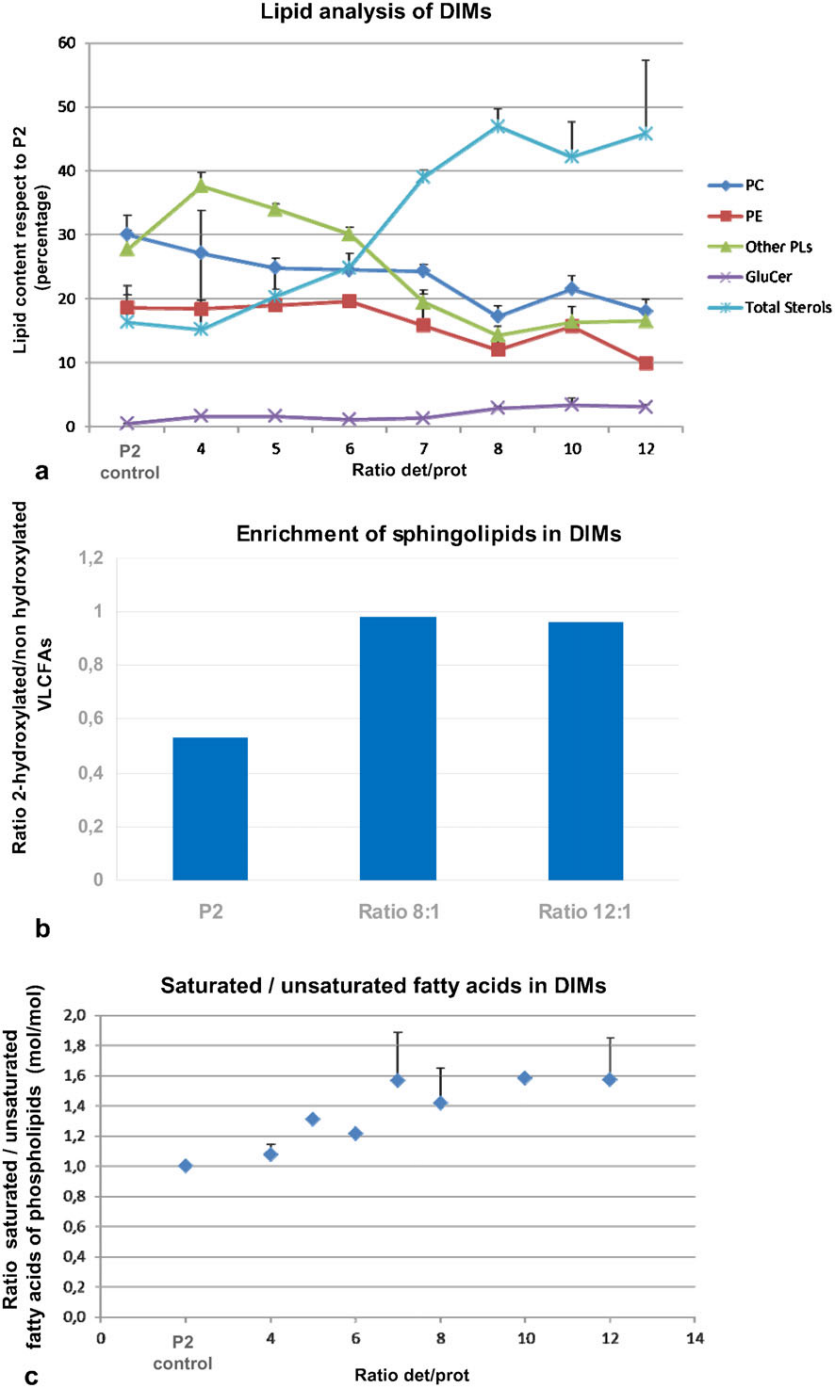
Lipid analysis. (a) HP-TLC analysis of DIMs of tobacco pollen tubes, using increasing detergent/protein ratios. The graph shows the percentages of different lipid species with respect to P2. The analysis refers to at least three independent preparations. A significant increase in total sterols and glucosylceramide (GluCer) is evident at ratio 8:1. The percentage remains constant at higher ratios of 10:1 and 12:1 (light blue and plum lines, respectively) while percentages of glycerophospholipids, such as phosphatidylcholine (PC, blue line), phosphatidylethanolamine (PE, red line) and other phospholipids (green line), decrease. (b) Analysis of hydroxylated/non-hydroxylated VLCFAs. The percentage increases significantly in DIMs with respect to P2 at detergent/protein ratios of 8:1 and 12:1 (mean of two experiments with similar values). (c) The graph shows the increased ratio of saturated/unsaturated phospholipid fatty acids in DIMs with respect to P2 at increasing detergent/protein ratios. The percentage increases up to ratio 7:1 and remains constant at ratios 8:1–12:1 (n = 3). Error bars indicate standard errors.

The close lateral association of sterols and sphingolipids is responsible for the most distinctive biochemical characteristic of membrane rafts, i.e. their insolubility in the presence of non-ionic detergents (Ahmed et al., 1997; Brown and London, 2000; London and Brown, 2000). We employed Triton X-100 to purify putative DIMs of the microsomal fraction (P2) of tobacco pollen tubes, using detergent/protein ratios ranging from 2:1 to 12:1. Triton-insoluble membranes floated as a single band at the interface between 15% and 30% in Optiprep density gradients at ratios comprised between 2:1 and 10:1 (supplementary material Fig. S1Aa, asterisk). At a detergent/protein ratio of 12:1 two floating bands were observed: one at the interface between 15% and 30% and a second band at higher density (supplementary material Fig. S1Ab, upper and lower band). The floating material obtained in experiments with different detergent/protein ratios was collected by ultracentrifuging and analysed for lipid profile. DIMs are known to be membrane microdomains rich in sterols and sphingolipids and with low concentrations of glycerophospholipids (Schroeder et al., 1994). DIMs obtained using ratios 4:1 to 12:1 in repeated experiments were analysed for lipid content and compared with P2 (Fig. 1a). Because the polypeptide profiles of the upper and lower bands obtained using the ratio 12:1 were similar (supplementary material Fig. S1B), the upper and lower bands were analysed together. Like DIMs isolated from animals (Simons and Sampaio, 2011) and plant somatic and cultured cells (Cacas et al., 2012), the quantity of sterols increased with the detergent/protein ratio, reaching a maximum at 8:1 (mean total lipids 46.9%), maintained at ratios of 10:1 and 12:1 (Fig. 1a, light blue line), whereas different species of glycerophospholipids (phosphatidylcholine, phosphatidylethanolamine and other phospholipids) decreased with respect to P2 (Fig. 1a, blue, brown and green lines, respectively). Previous studies indicated that DIMs isolated from plant PM are high in sphingolipids (Mongrand et al., 2004). In DIMs isolated from pollen tubes, the percentage of glucosylceramides (GluCer) also increased with the detergent/protein ratio and doubled with respect to P2 in DIMs obtained with ratios 8:1 and 12:1 (Fig. 1a, plum line). As an additional parameter to estimate the content of sphingolipids in DIMs, we considered the ratio of hydroxylated VLCFAs, more common in sphingolipids, and non-hydroxylated VLCFAs, typical of glycerophospholipids (Schroeder et al., 1994; Buré et al., 2014). The 2-hydroxylated/non-hydroxylated VLCFA ratio increased significantly with respect to P2 in DIMs purified with detergent/protein ratios of 8:1 and 12:1 (Fig. 1b). A further diagnostic criterion for DIM identification is the saturation of glycerophospholipid acyl chains (Schroeder et al., 1994; Mongrand et al., 2004). As shown for sterols and GluCer, the saturated/unsaturated fatty acid ratio increased with the detergent/protein ratio and reached higher values in DIMs purified with detergent/protein ratios of 8:1 and 12:1 (Fig. 1c).

Analysis of lipid profile showed that DIMs purified from tobacco pollen tubes using detergent/protein ratios of at least 8:1 met the criteria for membrane raft microdomains, suggesting that the physical basis for membrane raft formation (London, 2002) is maintained in pollen tubes. Since lipid (Fig. 1a–c) and polypeptide profiles did not change at a detergent/protein ratio of 12:1 (supplementary material Fig. S1B), we considered the ratio 8:1 for further experiments.

### Ultrastructural observation of DIMs

**Fig. 2.**
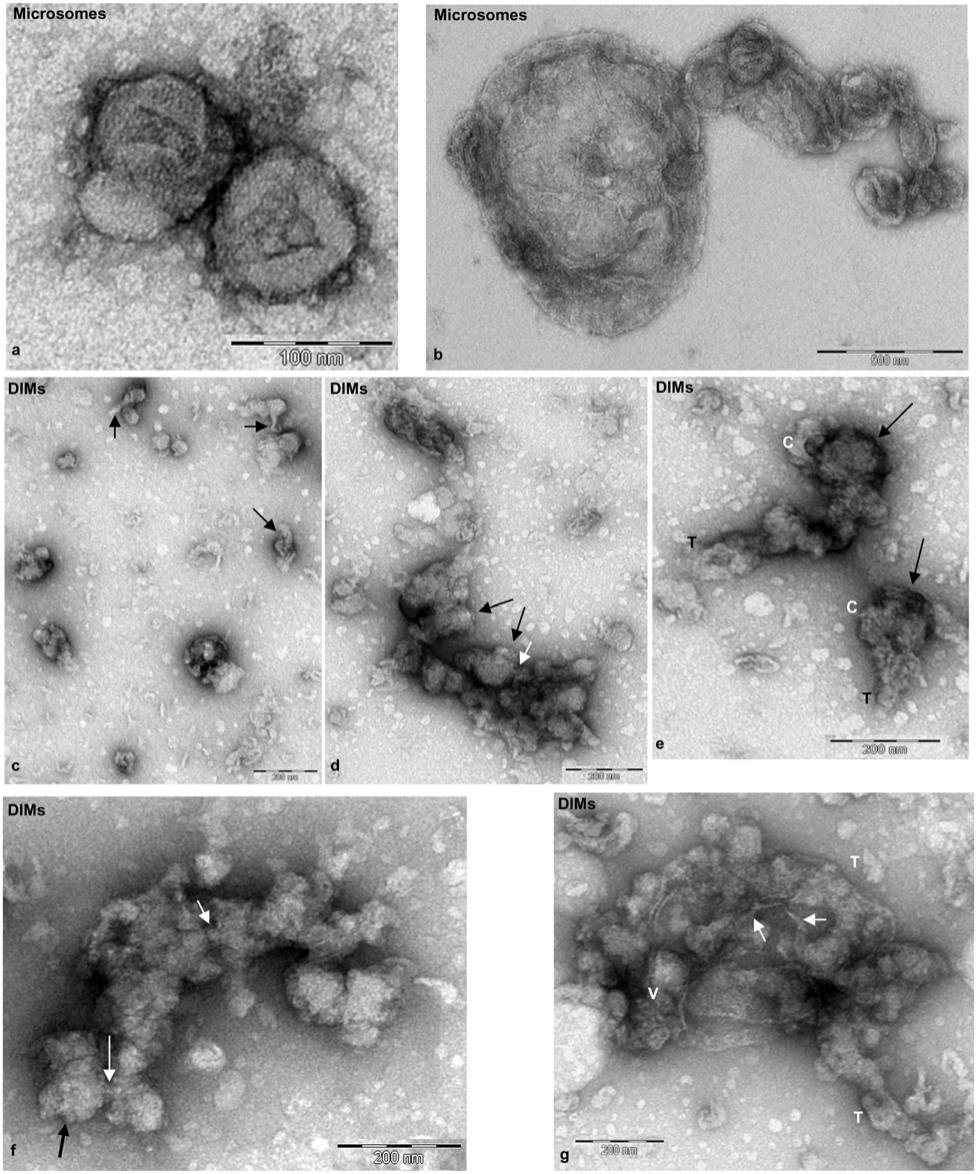
Transmission electron microscopy of fresh microsomes and DIMs. (a,b) P2 membranes appeared as regular vesicles having variable diameters ranging from 100 nm to 1 µm. (c–g) DIM membranes showed greater variability in size and shape than P2. Single vesicles (50–150 nm), with thin projections (c, arrows) alternate with vesicle clusters (d, arrows). Rounded cisternae (e,f, indicated as C) connected via tubular membranes (e, indicated as T; f, white arrows). Clusters of membranes comprising tubules (g, T) and clustered vesicles (V) connected by thin bridges (g, white arrows). Scale bars: 100 nm (a); 500 nm (b); 200 nm (c–g).

Freshly prepared microsomal membranes and DIMs were observed by transmission electron microscopy (TEM) after negative staining. Microsomes appeared as rounded vesicles or vesicle clusters with variable shapes and sizes (Fig. 2a,b). DIMs showed greater heterogeneity of membranous structures: vesicles with irregular shapes and diameters between 50 nm and 150 nm, often showing thin tubular projections (Fig. 2c, arrow), were observed as isolated elements or grouped into vesicle clusters (Fig. 2d). Larger membranes, consisting of rounded cisternae (Fig. 2e, indicated as C) and tubules (Fig. 2e, indicated as T), as well as clusters of irregular vesicles with rough surfaces (Fig. 2d,f, black arrows) connected by small tubules (Fig. 2d,f, white arrows) were also observed. Larger aggregates comprising tubules (Fig. 2e,g, indicated as T) and vesicle clusters (Fig. 2g, indicated as V) connected by thin bridges (Fig. 2g, white arrows) appeared to be enclosed in large membranes.

**Fig. 3.**
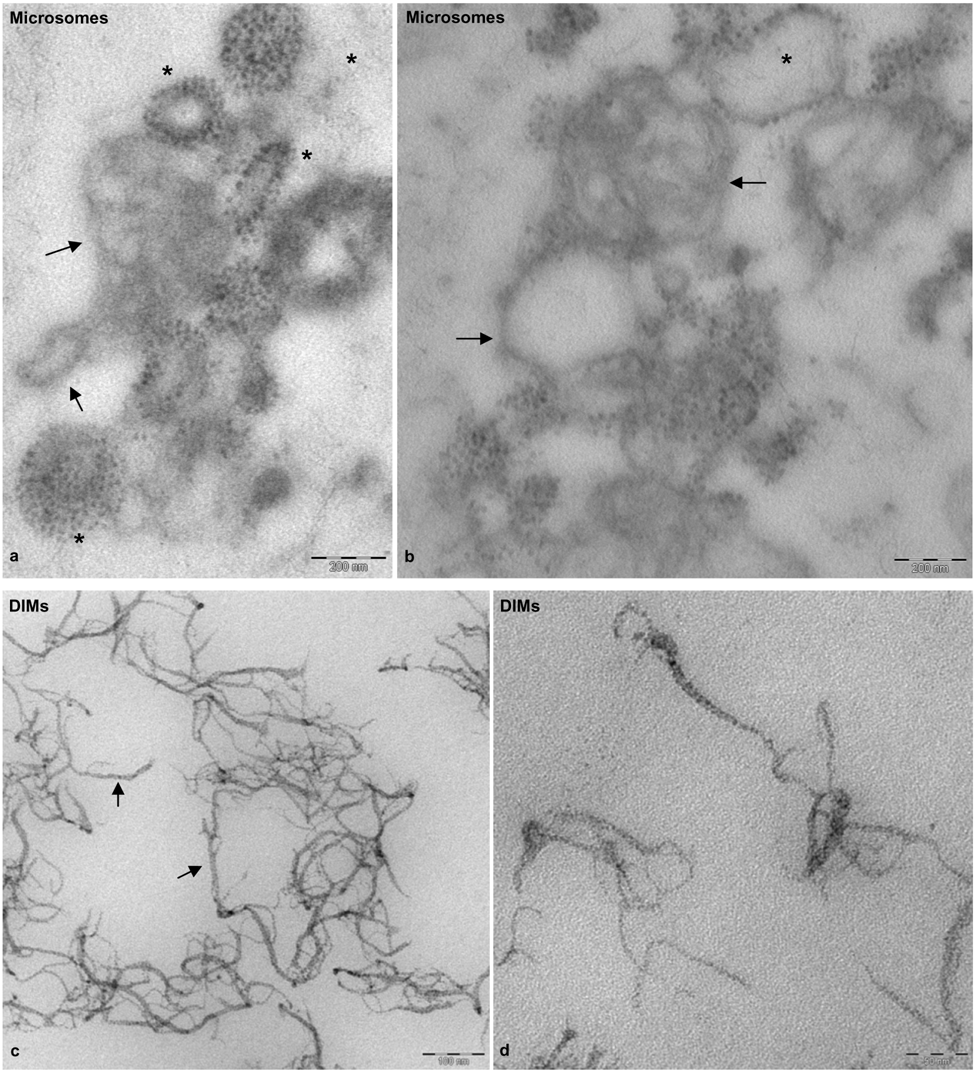
Transmission electron microscopy of fixed, embedded and sectioned microsomes and DIMs. (a,b) Sections of P2 membranes showing vesicles delimited by fainter membranes (arrows), seldom decorated with electron-dense particles (asterisks). (c,d) DIM membranes appearing as sharp, ribbon-like structures (arrows) with electrondense inclusions. Scale bars: 200 nm (a,b); 100 nm (c); 50 nm (d).

To analyse DIMs in more detail, pollen tube microsomes and DIMs were also observed by TEM after chemical fixation and sectioning. Microsomes showed vesicles with variable diameters, delimited by dimmer membranes (Fig. 3a,b, black arrows), sometimes decorated with electron dense particles (Fig. 3a,b, asterisks). Sharp ribbon-like membranes delimited DIMs (Fig. 3c) that mostly consisted of open structures (Fig. 3c), suggesting intrinsic membrane rigidity. Ribbon-like membranes were of variable width, ranging from 4 to 7 nm, as reported for DIMs purified from *Medicago truncatula* root PM (Lefebvre et al., 2007) and BY-2 cell PM (Mongrand et al., 2004). Moreover, electron dense material was observed in DIMs at higher magnification, suggesting the presence of protein inclusions (Fig. 3d).

### Protein analysis of DIMs

**Fig. 4.**
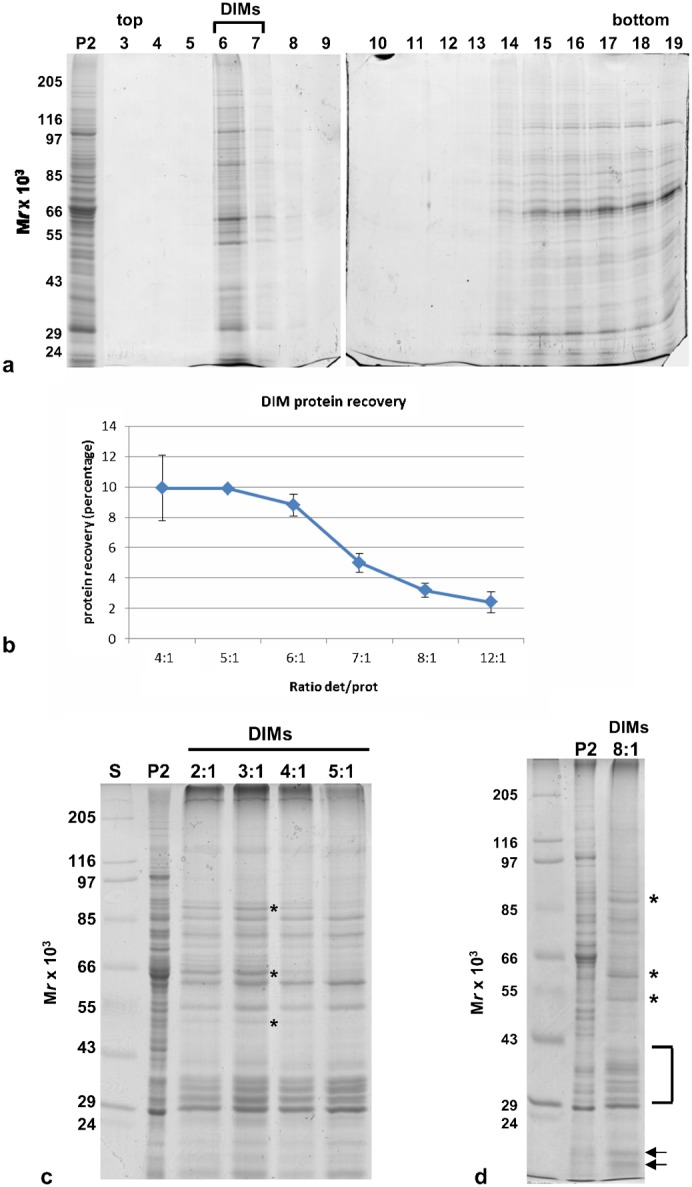
One-dimensional gel electrophoresis of DIMs. (a) Electrophoretic profile of fractions of the Optiprep density gradients. Fractions were run in two 10% polyacrylamide gels (10 slots for each gel). The floating material was recovered in fractions 6–7 in all experiments, irrespective of detergent/protein ratio. The same volume was loaded in each lane. (b) Graph showing the percentage of protein recovered in DIMs with respect to P2, at increasing detergent/protein ratios (3<*n*<11). Error bars indicate standard errors. (c) Electrophoretic profile of DIMs obtained with detergent/protein ratios of 2:1 to 5:1 compared with that of P2. 5 µg protein was loaded in each lane. Polypeptides indicated by the asterisks disappeared with increasing detergent/protein ratio. (d) Electrophoretic profile of DIMs obtained with a detergent/protein ratio of 8:1, compared with P2. Polypeptides indicated by the asterisks, arrows and brackets increased with respect to P2. 5 µg of protein was loaded in each lane.

DIMs obtained using increasing detergent/protein ratios were separated through discontinuous Optiprep density gradients. One-dimensional gel electrophoresis showed that DIMs were recovered in fractions 6–7 of the gradient, irrespective of detergent/protein ratio (Fig. 4a). Fractions 6–7 were pooled and centrifuged again to collect DIMs for further comparative studies. The percentage of proteins recovered from DIM fractions progressively declined with respect to P2 with increasing detergent/protein ratio, ranging from about 10% at a ratio of 4:1 to 3.5% and 2.5% at ratios of 8:1 and 12:1, respectively (Fig. 4b). This suggested that the increase in detergent gradually selected DIM-associated proteins. Electrophoretic analysis revealed qualitative differences in protein profiles, as some DIM polypeptides disappeared as the detergent/protein ratio rose from 2:1 to 5:1 (Fig. 4c, asterisks). The protein profile of DIMs, using a detergent/protein ratio of 8:1 in more than 10 experiments, reproducibly showed that a significant number of P2 polypeptides were not present in DIMs. Moreover, while the intensity of some bands seemed to be equivalent in the two samples, other polypeptides increased in DIMs with respect to P2 (Fig. 4d). A cluster of polypeptides comprised between 40 and 29 kDa was enriched in DIMs with respect to P2 (Fig. 4d, square bracket), as were polypeptides having apparent molecular masses of 85, 60 and 50 kDa (Fig. 4d, asterisks). Two polypeptides with a molecular mass of about 14 kDa were also enriched in the DIM fraction with respect to P2 (Fig. 4d, arrows).

**Fig. 5.**
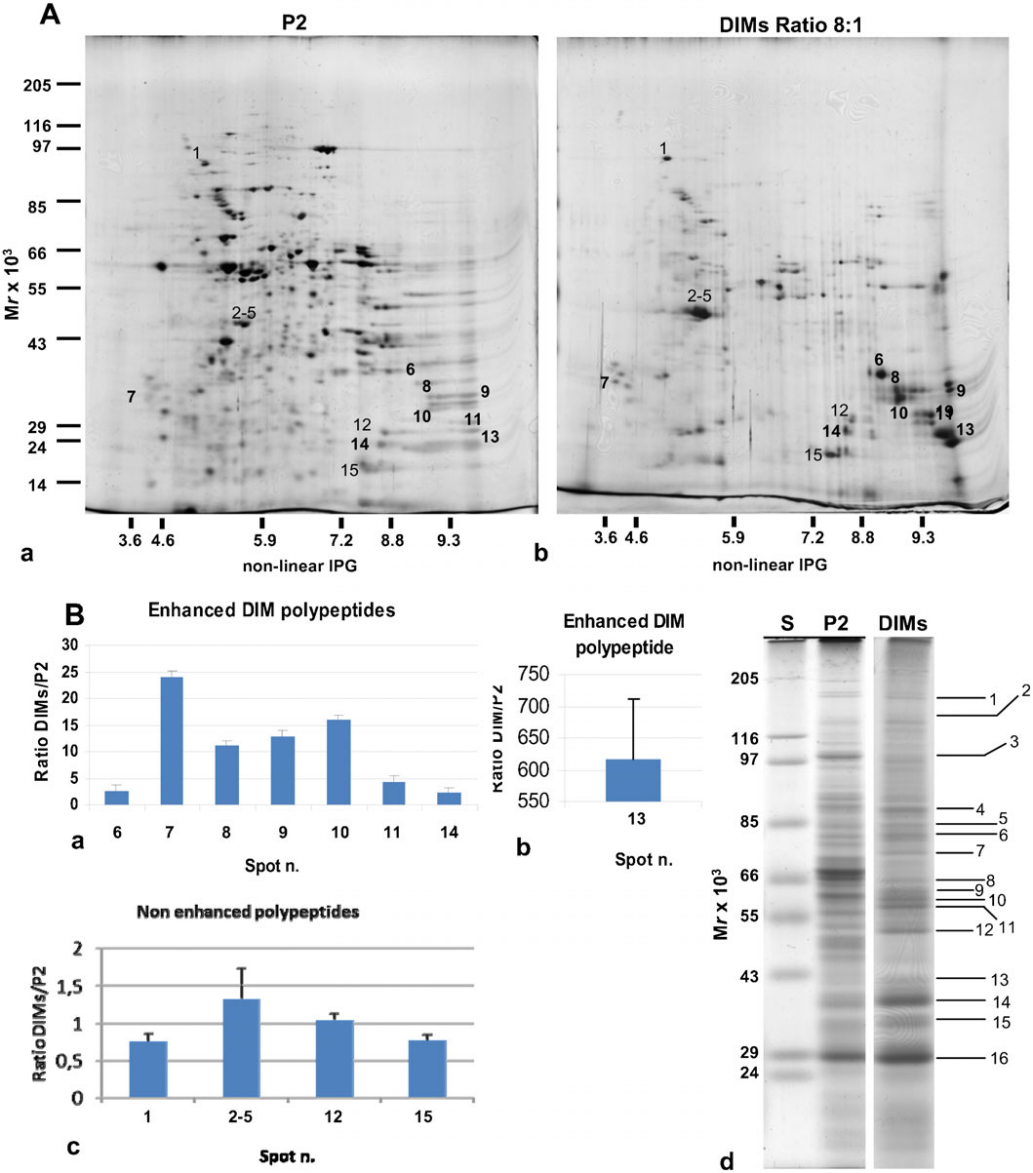
Two-dimensional maps of pollen tube microsomes and DIMs. (A) Representative silver-stained 2D gel electrophoresis of P2 (a) and DIMs (b) from tobacco pollen tubes (30 µg). The corresponding polypeptides, identified by MS-MS analysis, are indicated with normal (not enhanced) and bold (enhanced) numbers. (B) Graphs showing the ratio of enhanced (a,b) and non-enhanced (c) polypeptides identified by MS-MS analysis in DIMs and P2. Error bars indicate standard errors (n = 2). (d) One-dimensional preparatory electrophoresis of pollen tube DIMs (detergent/protein ratio 8:1). 30 µg protein was loaded in the lane. P2 and DIMs were prepared in the same experiment and run in different gels. Polypeptides identified by MS-MS analysis are indicated.

To analyse the polypeptide profile of DIMs in more detail, membranes purified at a detergent/protein ratio of 8:1 were resolved by two-dimensional (2D) gel electrophoresis. Repeated experiments were performed to find a good compromise between the amount of protein loaded (14 µg–50 µg) and IEF separation conditions. We found that 30 µg was the quantity of protein that met our needs. Representative 2D maps of P2 and DIM proteins were analysed by PD-Quest (Fig. 5Aa,b). The study showed a mean of 479 spots for P2, reproducibly separated for each of the two replicates, indicating a mean of 197 spots (35% of total protein of P2) for DIMs. Of these, 70% were enhanced with respect to P2.

### Protein identification

To unravel the role of membrane rafts in pollen tubes, DIM polypeptides were excised from 2D gels and subjected to MALDI-TOF MS and/or LC-MS/MS analysis. The enhanced and non-enhanced DIM proteins identified are indicated in Fig. 5A,B, with bold and normal numbers, respectively. Pollen tube DIMs, isolated from the microsomal fraction, showed unusual features compared to those isolated from plant somatic cells. Five actin sequences were specifically identified in the cluster of spots 2–5 (Fig. 5Bc; Table 1). Although the actin clusters of two DIM preparations seemed to be concentrated differently with respect to P2 by PD-Quest analysis (supplementary material Fig. S2, spots 2-5), western blot assays on equal amounts of P2 and DIMs from three purification experiments showed that actin was not significantly enriched in DIMs with respect to P2 (supplementary material Fig. S3A,B). Moreover, although several polypeptides were observed in the gels and five actin sequences were identified (Fig. 5Bc; Table 1), ClustalW analysis revealed a single actin protein (supplementary material Fig. S4A), suggesting that the spots observed in the cluster may be the consequence of post-translational modifications. Other proteins specifically present in pollen tube DIMs included the mitochondrial stress-responsive protein, prohibitin (Fig. 5Ab; Table 1, spot 9). The 2D gel separated a polypeptide (Fig. 5Ab, spot 13) that was 95% identical (100% query cover) to *Nicotiana tabacum* prohibitin (accession AAC49690) and a further member of the same protein family was identified in spot 9 (Fig. 5Ab; Table 1, PHB2), the latter being 50% identical to the former. PD-Quest analysis showed that both prohibitin family members were enriched in DIMs with respect to P2 (Fig. 5Ba,b; supplementary material Fig. S2 and Fig. S4C).

**Table 1.**
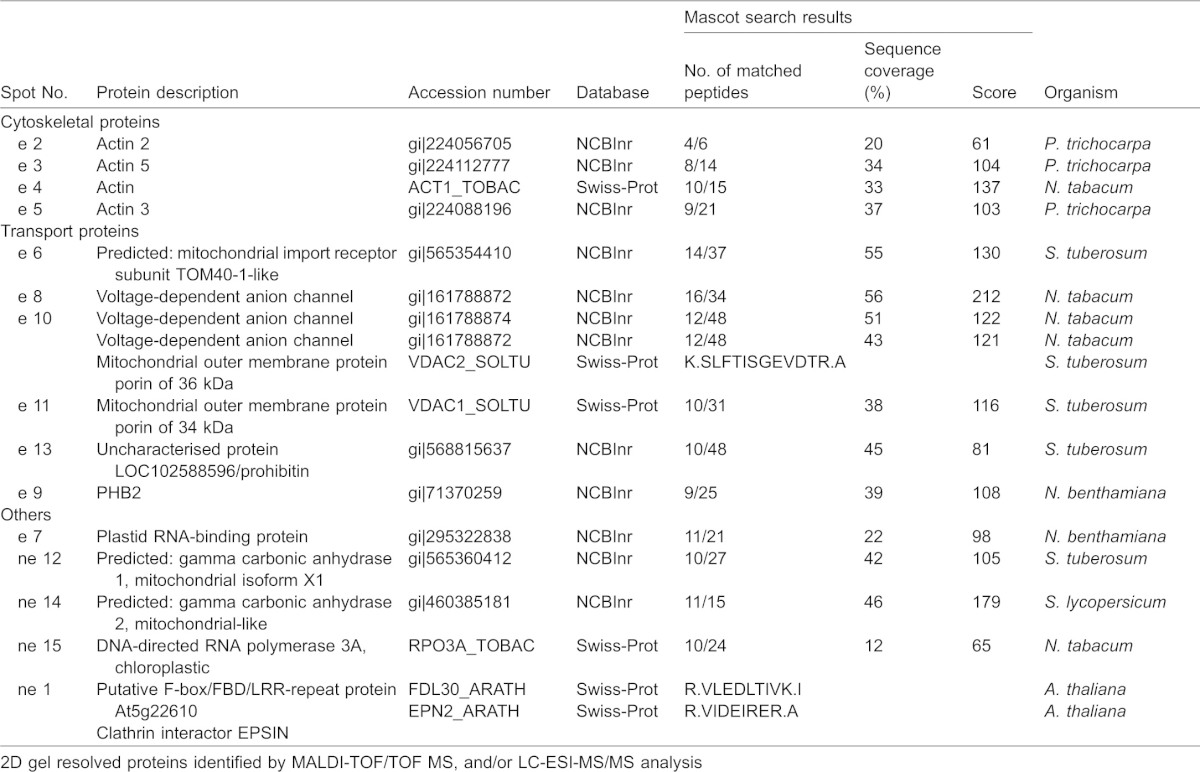
Enriched (e) or non-enriched (ne) proteins in DIMs

Other proteins enriched in DIM fractions included VDACs (Table 1, spots 8, 10, 11 in Fig. 5Ab; Fig. 5Ba), previously identified in DIMs isolated from *Medicago truncatula* PM (Lefebvre et al., 2007). ClustalW analysis of the four VDAC sequences suggested the presence of three VDAC isotypes (gi|161788872, gi|161788874/VDAC2_SOLTU, gi|161788876) (supplementary material Fig. S4B). Interestingly, the same VDAC (gi|161788872) was found in spots 8 and 10 (Fig. 5Ab; Fig. 5Ba; Table 1), suggesting post-translationally modified versions of the same polypeptide. Other enhanced proteins of pollen tube DIMs included a polypeptide of the mitochondrial outer membrane, porin (Table 1; Fig. 5Ab, spot 10) and additional mitochondria and chloroplast polypeptides, seldom found in plant raft domains (Table 1; spots 7, 12, 14, 15, supplementary material Fig. S2) (Borner et al., 2005; Mongrand et al., 2004; Lefebvre et al., 2007). The clathrin interacting protein, epsin, and a putative F-box FBD/LRR-repeat protein were also identified in spot 1 (Fig. 5Ab; Table 1) as non-enriched proteins of pollen tube DIMs (Fig. 5Ab; Fig. 5Bc; supplementary material Fig. S2).

As many integral membrane proteins cannot effectively be resolved by 2D gels (Rabilloud, 2009), DIM polypeptides were also identified by MALDI-TOF MS analysis after separation by preparatory 1D gel electrophoresis (Fig. 5Bd; Table 2). This study confirmed the presence of actin (Fig. Bd; Table 2, band 12) as an unusual component of pollen tube DIMs and identified additional polypeptides not found in DIMs isolated from other plant cells. Intriguingly, a member of the SAC domain phosphoinositide phosphatase family (SAC6), involved in regulating phosphoinositide production and in turn, cytoskeleton reorganisation (Zhong et al., 2005) and cell wall deposition in response to stress (Thole et al., 2008), was also specifically identified (band 7), suggesting that membrane rafts may indeed be platforms to translate external stimuli into specific patterns of pollen tube growth. Enzymes, such as S-adenosylmethionine synthase and PMT3 methyltransferase, were also revealed as DIM-enriched proteins (Fig. 5Bd; Table 2, band 4 and 11, respectively), suggesting that DIMs could be sites for coupling the S-methylmethionine cycle with methylation pathways (Ranocha et al., 2001).

**Table 2.**
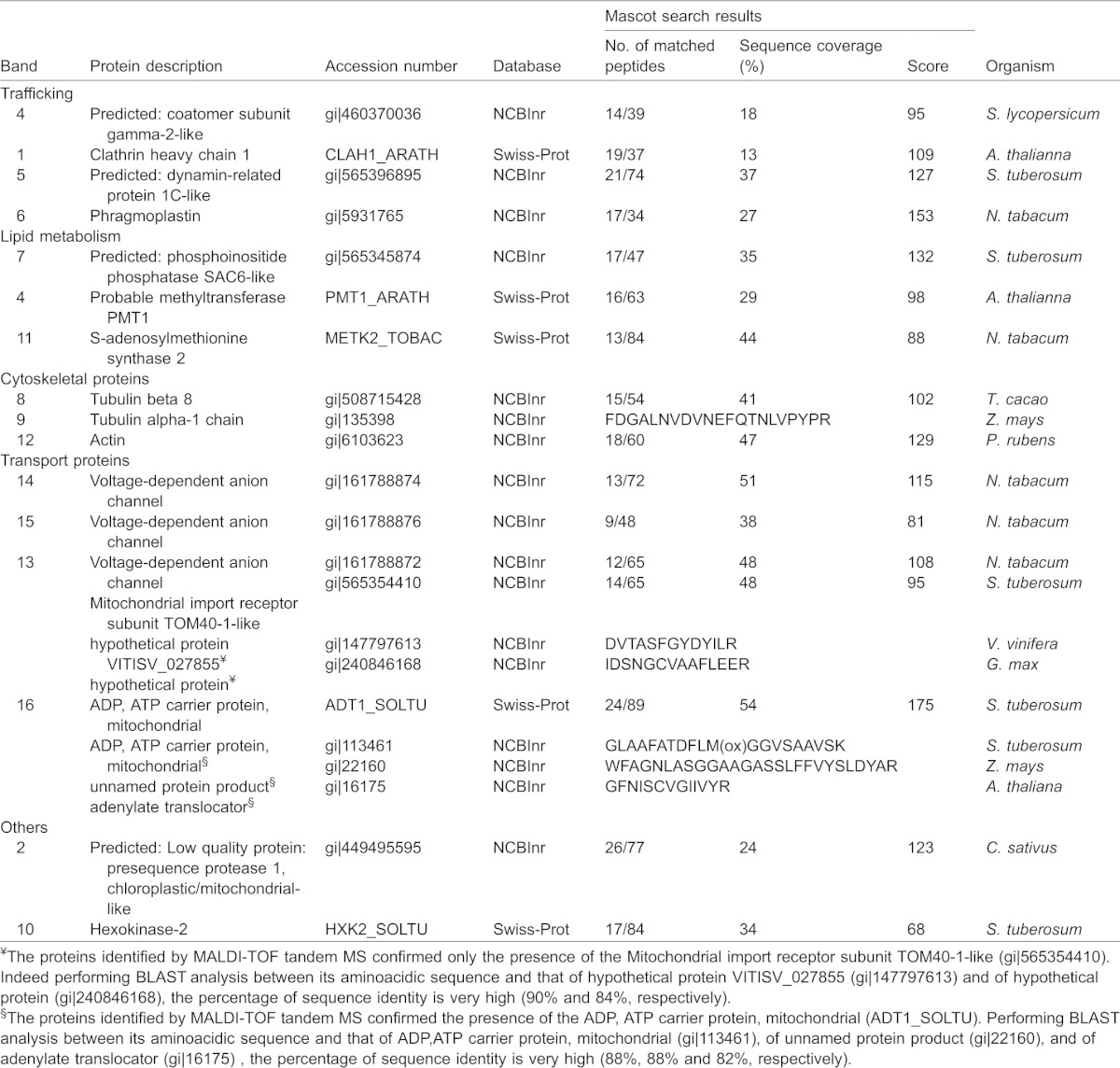
DIM proteins from 1D gel identified by MALDI-TOF/TOF MS, and/or LC-ESI-MS/MS analysis

One dimensional gel analysis further confirmed the presence of VDACs (Fig. 5Bd; Table 2, bands 13, 14, 15) and revealed that α-tubulin and β-tubulin were also associated with DIMs (Fig. 5Bd; Table 2, bands 8, 9), as already reported in plant somatic cells (Mongrand et al., 2004; Borner et al., 2005; Lefebvre et al., 2007). This led us to postulate that besides actin, microtubules may contribute to the organisation of membrane raft architecture/clustering or that DIMs may regulate actin and microtubule function in pollen tubes. Protein study by one-dimensional gel also identified additional proteins that regulate membrane trafficking, such as coatomer subunit gamma 2 (Fig. 5Bd; Table 2, band 3), clathrin heavy chain isoform 5 (Fig. 5Bd; Table 2, band 1) and dynamin-like proteins involved in vesicle fission (Fig. 5Bd; Table 2, bands 5, 6).

**Fig. 6.**
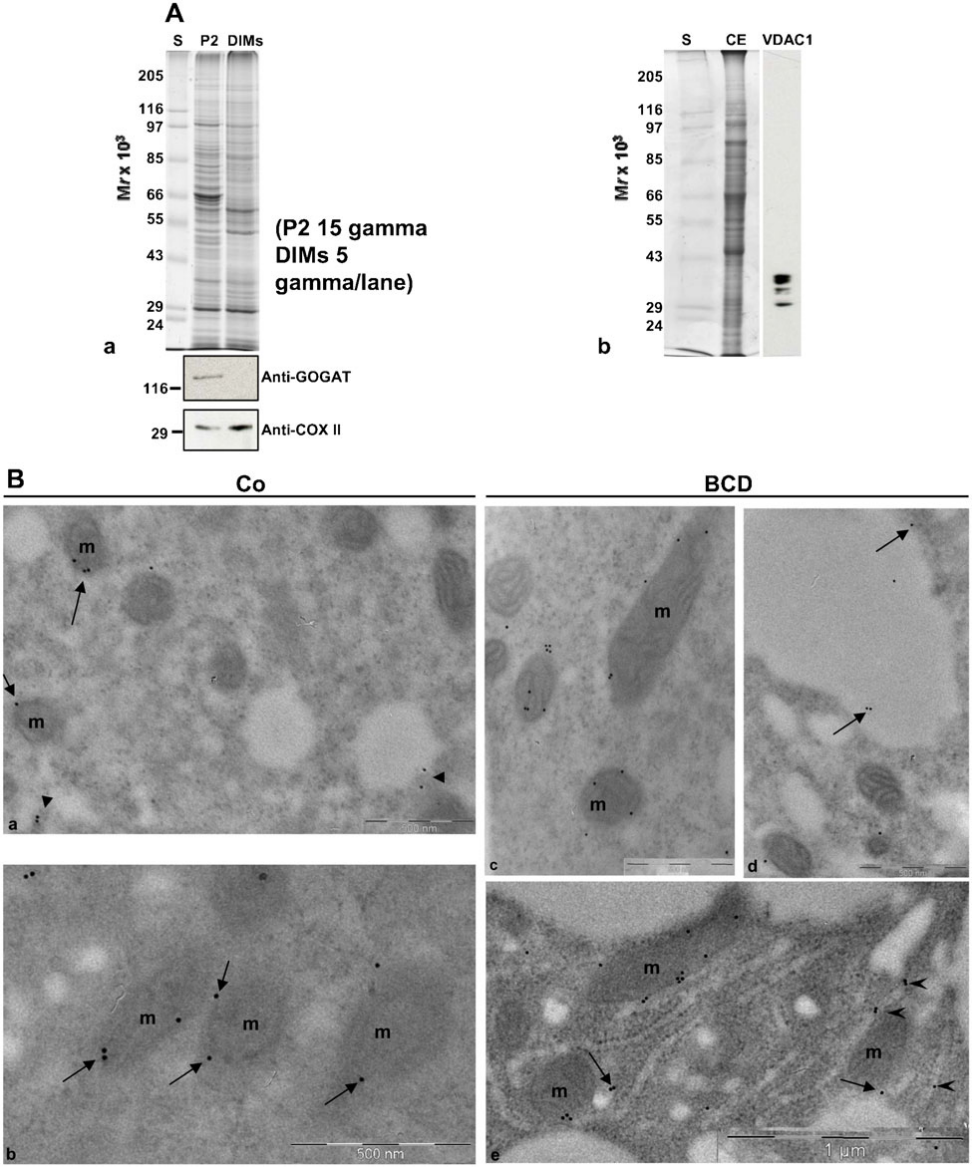
DIM characterisation. (A) Contamination of DIMs by plastid and mitochondrial membranes. (a) Anti-plastid (GOGAT) and anti-mitochondria (COXII) markers were probed on P2 (15 µg) and DIMs (6 µg). (b) The specificity of the anti-VDAC antibody probed on pollen tube crude extract. The antibody recognised four VDAC bands, identified by MS spectrometry. (B) Immunolocalisation of VDACs in pollen tubes. (a,b) In control pollen tubes the anti-VDAC antibody labelled the mitochondrial (mitochondria are indicated as m) outer membrane (arrows) and cytoplasmic vesicles (a, arrowheads). (c–e) In BCD-treated pollen tubes the anti-VDAC antibody still stained the mitochondrial outer membrane and vesicles (e, arrows), but also vacuoles (d, arrows) and ER (e, arrowheads).

The MALDI-TOF MS and LC-MS/MS analysis identified polypeptides from mitochondria and plastids, presumably due to aspecific contamination of mitochondria/plastid proteins adhering to DIMs. Since isolation of pollen tube DIMs was carried out from the microsomal fraction, the presence of mitochondria and plastid proteins could otherwise depend on the presence of mitochondrial/plastid DIMs. To answer this question western blot analysis was performed on P2 and DIMs, using antibodies against the mitochondria and plastid markers cytochrome-c oxidase subunit II (COXII) and glutamine oxoglutarate aminotransferase (GOGAT), respectively. The anti COXII antibody recognised a band with a molecular mass of about 30 kDa both in P2 and DIMs (Fig. 6Aa), suggesting that mitochondria were present in the microsomal fraction and that mitochondrial DIMs were present in our preparation. On the contrary, while plastids appeared to be present in P2, no reaction was detected in DIMs (Fig. 6Aa), supporting the idea that plastid membranes do not contaminate DIMs and that the presence of plastid proteins could be due to spurious contamination by proteins that are solubilised during the DIM isolation procedure.

Among mitochondrial proteins enhanced in DIMs, MALDI-TOF MS and LC-MS/MS analysis identified at least four mitochondrial VDACs/porins, that were also found in DIMs isolated from *Medicago truncatula* PM (Lefebvre et al., 2007). To verify the localisation of VDACs in pollen tubes we performed immunolocalisation experiments using a polyclonal antibody against VDACs. The specificity of the antibody was previously tested on the pollen tube crude extract (Fig. 6Ab). According to MALDI-TOF MS and LC-MS/MS analysis, the anti-VDAC antibody recognised bands between 40 kDa and 29 kDa. Immunogold-labelling experiments revealed that VDACs localised on the outer mitochondrial membrane (Fig. 6Ba,b, arrows) and decorated cytoplasmic vesicles (Fig. 6Ba, arrowheads). Control experiments in which the primary antibody was omitted showed a few, occasional gold particles dispersed in the cytoplasm (supplementary material Fig. S5b), whereas cytoplasmic organelles did not stain (supplementary material Fig. S5a–c).

### β-cyclodextrin dramatically inhibited the pollen tube growth and affected both DIMs and pollen tube morphology

To validate our data showing lipid microdomains in Angiosperm pollen tubes, we investigated the effect of different concentrations of β-cyclodextrin (BCD) on DIMs and on pollen tube morphology. The BCD is known to extract sterols from membranes and to destroy lipid rafts in different cell types (Ohtani et al., 1989; Ilangumaran and Hoessli, 1998; Roche et al., 2008). In order to observe the effects of sterol deprivation on isolated DIMs and on pollen tube ultrastructure, tobacco pollen tubes were incubated with 8 mM or 16 mM BCD. Germination assays were performed to identify the best BCD concentration and incubation time. We found that incubation with both 8 mM and 16 mM BCD for 2 h and 30 min, dramatically inhibited pollen tube elongation (supplementary material Fig. S6a–d; *P*<0.001), suggesting that sterols play a key role in polarised growth.

**Fig. 7.**
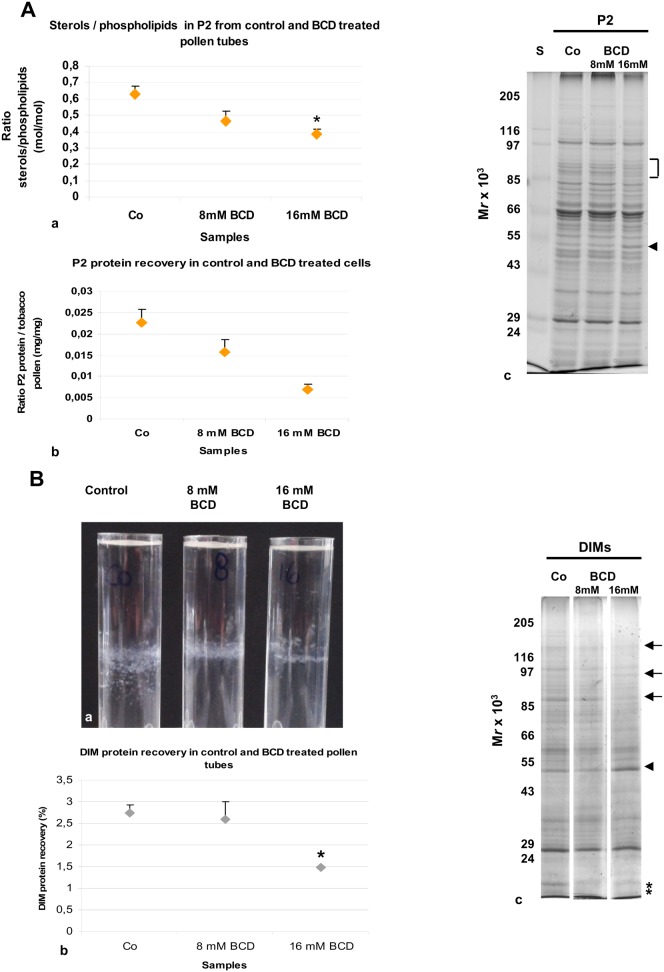
Effect of BCD on DIM isolation. (A) Effect of BCD on microsomal fraction. (a) Lipid analysis revealed a decrease in sterol content with 8 mM and 16 mM BCD treatment, with respect to control cells. (b) Less protein was recovered in P2 when pollen tubes were incubated with 8 mM and significantly less after incubation with 16 mM BCD with respect to control. (c) Electrophoretic analysis showed changes in the polypeptide pattern of 16 mM BCD-treated pollen tubes P2 (square bracket indicates decreasing polypeptides, arrowhead indicates enhanced polypeptide), with respect to control. (B) Effect of BCD on DIMs. (a) The amount of material in the floating band decreased when pollen tubes were incubated with BCD. The behaviour of DIMs also changed from fine particles in control cells to lamellae in 16 mM BCD-treated samples. (b) A significant decrease in DIM protein recovery was detected in 16 mM BCD-treated pollen tubes. Error bars indicate standard errors. (c) Electrophoretic analysis revealed changes in the electrophoretic pattern in DIMs isolated from 16 mM BCD treated pollen tubes (arrows and asterisks indicate decreasing polypeptides, the arrowhead indicates the increased polypeptide). Control, 8 mM and 16 mM DIMs were run in the same gel, but in non-adjacent lanes.

To analyse the effect of sterol extraction on pollen tubes and DIMs, cells grown in the presence of 8 mM and 16 mM BCD for 2 h and 30 min were processed for DIM isolation. β-cyclodextrin profoundly affected P2 and DIMs. Lipid analysis of P2 from control and treated cells showed a significant decrease in the sterols/PLs ratio in 16 mM treated cells, with respect to control (Fig. 7Aa; *P*<0.05). In addition, protein content recovered in P2 progressively decreased in pollen tubes grown with BCD and a significant reduction was observed with 16 mM BCD over three independent experiments (Fig. 7Ab; *P*<0.05), suggesting that BCD alters protein redistribution between microsomal and soluble fractions. The 1D electrophoretic analysis of P2 prepared from control and BCD treated cells revealed slight differences in the polypeptide pattern between the control and 16 mM treated pollen tubes (Fig. 7Ac; arrowhead and square bracket). After incubation of P2 with triton X 100, a reduction in the floating band was observed with 16 mM BCD, with respect to control over three independent experiments (Fig. 7Ba). Analysis of DIM protein recovery also showed a significant reduction with 16 mM BCD treatment (Fig. 7Bb; *P*<0.05), suggesting that sterol depletion dramatically altered DIM integrity. Polypeptide profile analysis by 1-D gel did not show any change in the electrophoretic pattern of 8 mM BCD DIMs with respect to control, whereas with 16 mM BCD treatment, a reduction in polypeptides over 85 kDa (Fig. 7Bc, see arrows) and below 24 kDa was observed (Fig. 7Bc, see asterisks); on the other hand, a polypeptide with molecular mass of 50 kDa appeared to increase (Fig. 7Bc, see arrowhead), suggesting that sterol depletion also induced a redistribution of DIM polypeptides. As a matter of fact, immunogold-labeling experiments, using the anti-VDAC antibody in 16 mM BCD treated pollen tubes revealed changes in the localisation of these proteins with respect to control. While the staining of mitochondrial outer membrane (Fig. 6Bc,e) and vesicles were not affected (Fig. 6Be, arrows), mislocalisation of VDACs to vacuoles (Fig. 6Bd, arrow) and to the ER was observed (Fig. 6Be, arrowheads).

Due to the effect of 16 mM BCD on DIM protein recovery and composition, subsequent experiments were carried out in the presence of 16 mM BCD.

**Fig. 8.**
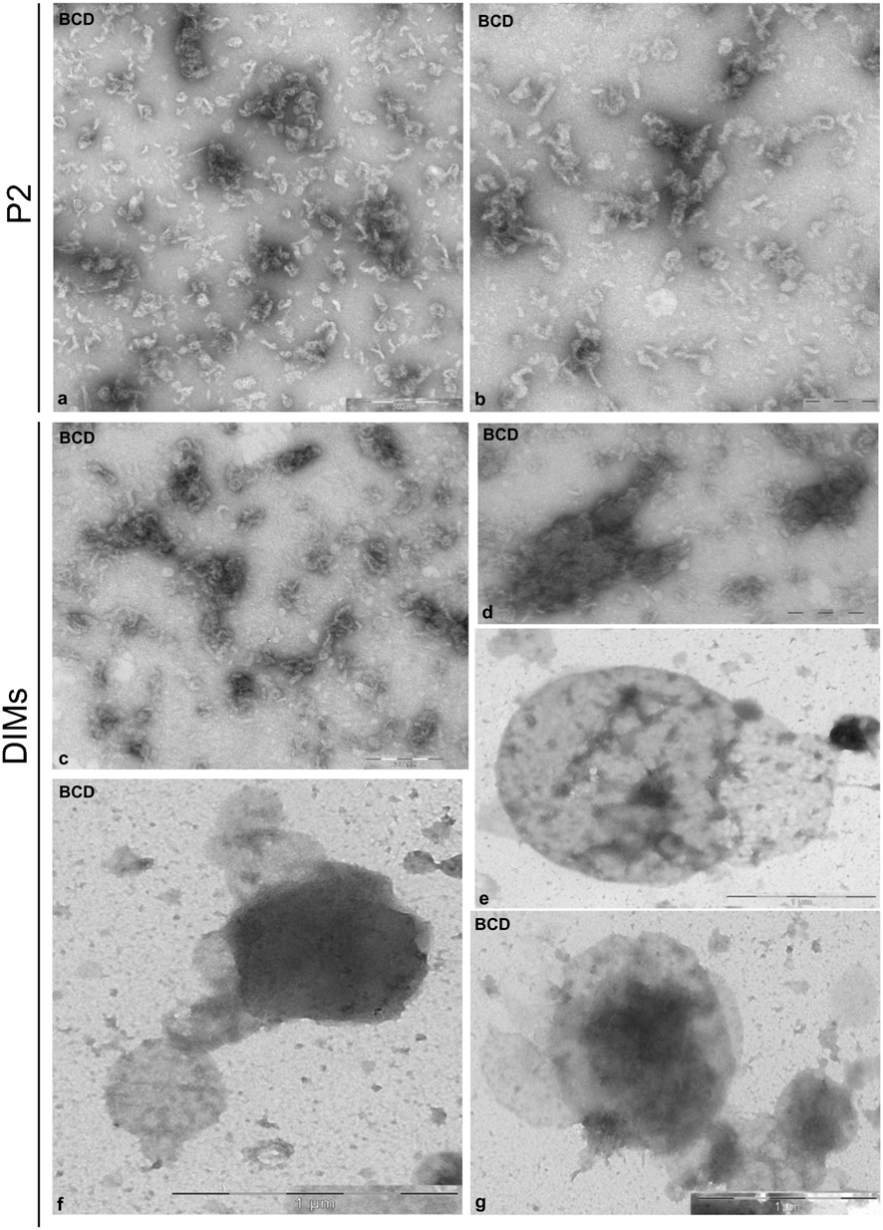
Effect of BCD on P2 and DIM ultrastructure. (a,b) The microsomal fraction appeared to be made up of membrane fragments, instead of organelles. (c–g) Isolated DIMs showed as clusters of membrane fragments (c,d) or as smooth vesicles with diameters of 0.5–1 µm (e–g). Scale bars: 200 nm (b-d); 500 nm (a); 1 µm (g,f).

Because control DIMs appeared as small particles, whereas DIMs from 16 mM BCD treated cells appeared as lamellae (Fig. 7Ba), it may be postulated that BCD treatment also dramatically altered DIM ultrastructure. To observe the effect of BCD in greater detail, P2 and DIMs freshly isolated from pollen tubes grown in the presence of 16 mM BCD were observed by transmission electron microscopy and compared with control samples. A profound effect of BCD was observed in P2 isolated from treated cells. While control P2 was characterised by distinct organelles (Fig. 2a,b), fragmented membranes were observed in the BCD microsomal fraction (Fig. 8a,b), suggesting that sterol depletion induces organelle fragility. Dramatic changes were also observed in DIM ultrastructure compared to controls (Fig. 2): the floating material consisted of membrane fragments instead of well-defined vesicles or vesicle clusters (Fig. 8c,d) and smooth compartments with diameter over 500 µm (Fig. 8e–g).

These results show that sterol depletion decreases DIM recovery and affects DIM ultrastructure, thus sustaining the presence of membrane microdomains in tobacco pollen tubes.

### Localisation of sterols and ratiometric live imaging of di-4-ANEPPDHQ in growing pollen tubes

**Fig. 9.**
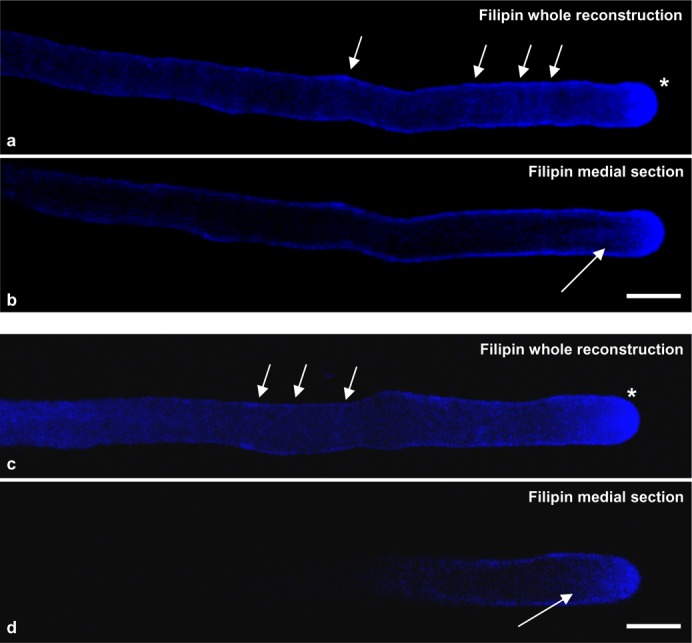
Sterol localisation. (a,c) Whole reconstructions. Sterols are present on the PM, where a ring-like distribution was often evident (arrows). Sterol-rich domains were especially concentrated in the apex PM (asterisks). (b,d) Medial sections. Sterols also accumulate in clear zone vesicles (arrows). Scale bars: 10 µm.

DIMs purified from tobacco pollen tubes had a high sterol content. In order to localise sterol-rich membranes, pollen tubes were stained with filipin, that is known to bind sterols (Muller et al., 1984) in organisms as different as animals (Reid et al., 2004), fungi (Martin and Konopka, 2004) and plants (Grebe et al., 2003; Ovecka et al., 2010; Boutté et al., 2010). As growing pollen tubes incubated with filipin immediately stopped growing (data not shown), cells were fixed and then stained with the probe. Observation of a number of pollen tubes showed that sterol-rich domains were present on the PM, where a ring-like distribution was often evident, reminiscent of AGPs and pectins during pulsed growth (Li et al., 1994; Li et al., 1996) (Fig. 9a,c, arrows). In addition, sterol-rich domains were especially concentrated in the PM of the whole apex (Fig. 9a,c, asterisks) and in vesicles accumulating in the clear zone (Fig. 9b,d, arrows), suggesting that sterol-rich membrane domains could be involved in polarised secretion and in asymmetric distribution of proteins and lipids along the pollen tube, as a prerequisite for sexual reproduction and seed set.

**Fig. 10.**
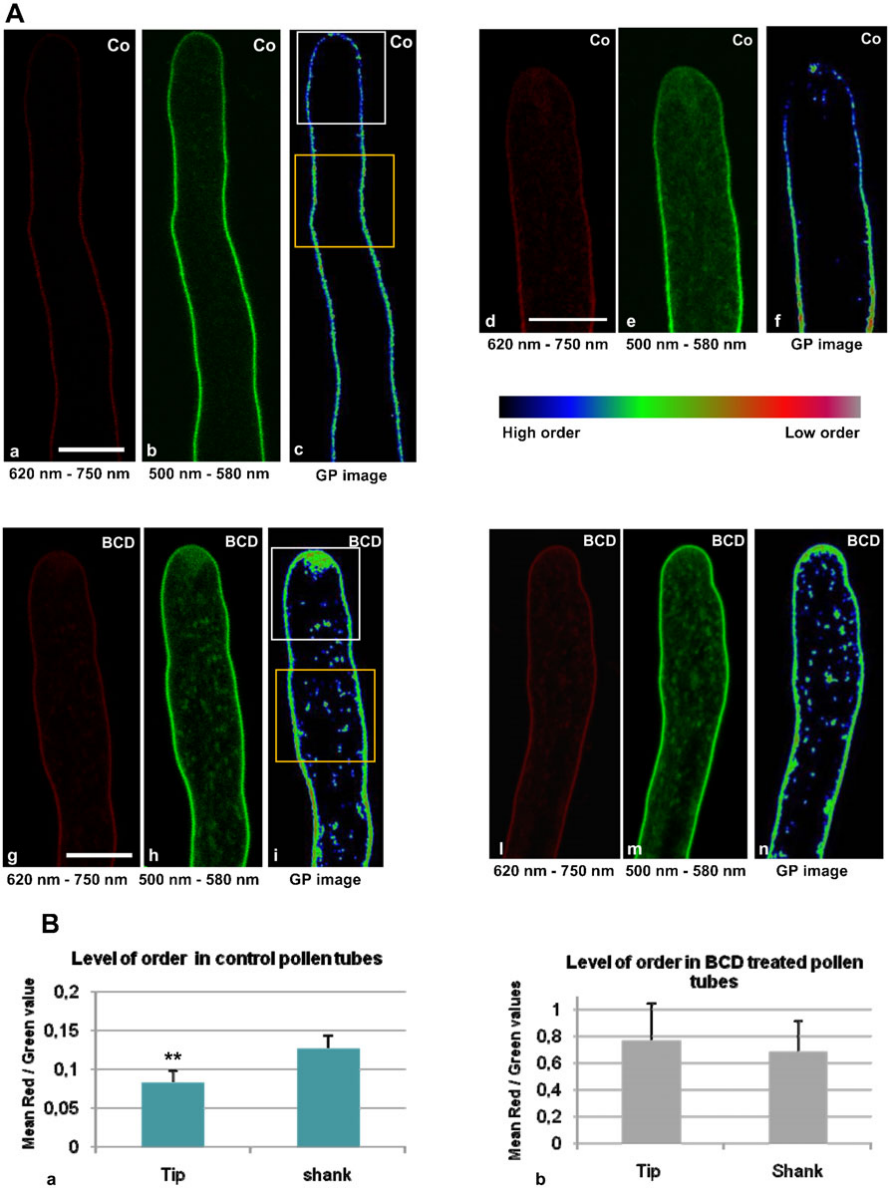
Ratiometric live imaging of di-4-ANEPPDHQ in growing pollen tubes. (A) Higher and lower membrane order distribution in control and BCD treated pollen tubes. (a–f) Red (a,d) and green (b,e) channels of control pollen tubes. GP images (c,f) revealed a higher membrane order in the tip region than in the shank. (g–i,l–n) In BCD-treated pollen tubes both the red (g,l) and green (h,m) channel intensities were uniform in tip and shank. GP images (i,n) showed loss of asymmetry in higher-order membrane distribution along pollen tube. Scale bars: 10 µm. (B) Ratio of ratios. (a) Red/green values calculated in the tip (white ROI) and shank (yellow ROI) of control pollen tubes. The mean red/green ratio in the tip was significantly lower than in the shank (n = 12). (b) Red/green ratios in the tip (white ROI) and shank (yellow ROI) of BCD-treated pollen tubes. Mean red/green ratios in tip and shank were equal and higher than in controls (n = 9). Error bars indicate standard errors. ** indicates P<0.001.

To study the relation between sterol distribution and the presence of membrane microdomains in live cells, growing pollen tubes were stained with di-4-ANEPPDHQ, a styryl dye with distinct green and red emission spectra, in order to distinguish L_o_ and L_d_ phases, respectively (Jin et al., 2005; Jin et al., 2006; Owen et al., 2006). Dye loading was conducted for 1 min in control cells and after incubation with 16 mM BCD, after which the cells were imaged at different times over 20 min. The probe only stained PM in the first 10 min after dye removal (Fig. 10Aa–c), whereas staining of vesicles in the clear zone was also observed after 15–20 min, both in control and BCD treated-cells (Fig. 10Ae,h). Ratiometric live imaging of di-4-ANEPPDHQ detected membrane order levels in growing pollen tubes. Emission fluorescence intensities in the two channels, (620–750 nm, red and 500–580 nm, green), were used to generate ratiometric, pseudo colored General Polarisation (GP) images in which red and green indicated lower and higher orders, respectively (Fig. 10A). In control cells, ratiometric analysis showed asymmetric distribution of the two phases along growing pollen tubes (Fig. 10Aa–c,d–f), revealing significantly higher ordered membrane in the apical region than in the shank (Fig. 10Ba; *P*<0.001, n = 9). On the contrary, BCD-treated pollen tubes revealed a general increase in low membrane order in the PM (Fig. 10Bb) and the asymmetry between the tip and shank disappeared (Fig. 10Ag–i,l–n; Fig. 10Bb, n = 12). In the clear zone, fluorescent vesicles appeared about 15 min after the pulse, both in control and BCD-treated pollen tubes, suggesting that stained PM was endocytosed and recycled by cytoplasmic membrane trafficking (Parton et al., 2001; Moscatelli et al., 2007; Zonia and Munnik, 2008; Onelli and Moscatelli, 2013). In control cells the clear zone vesicles appeared to be formed by high ordered membranes (Fig. 10Ad–f), in BCD-treated cells apical localised vesicles were characterised by lower ordered membranes (Fig. 10Ag–i).

All together these results showed a polarised distribution of membrane microdomains along the pollen tube PM, with higher ordered membranes localised in the tip region rather than in the shank. The asymmetric localisation of ordered membranes also matched a higher presence of sterols and was abolished by BCD.

### β-cyclodextrin affected tobacco pollen tube morphology

**Fig. 11.**
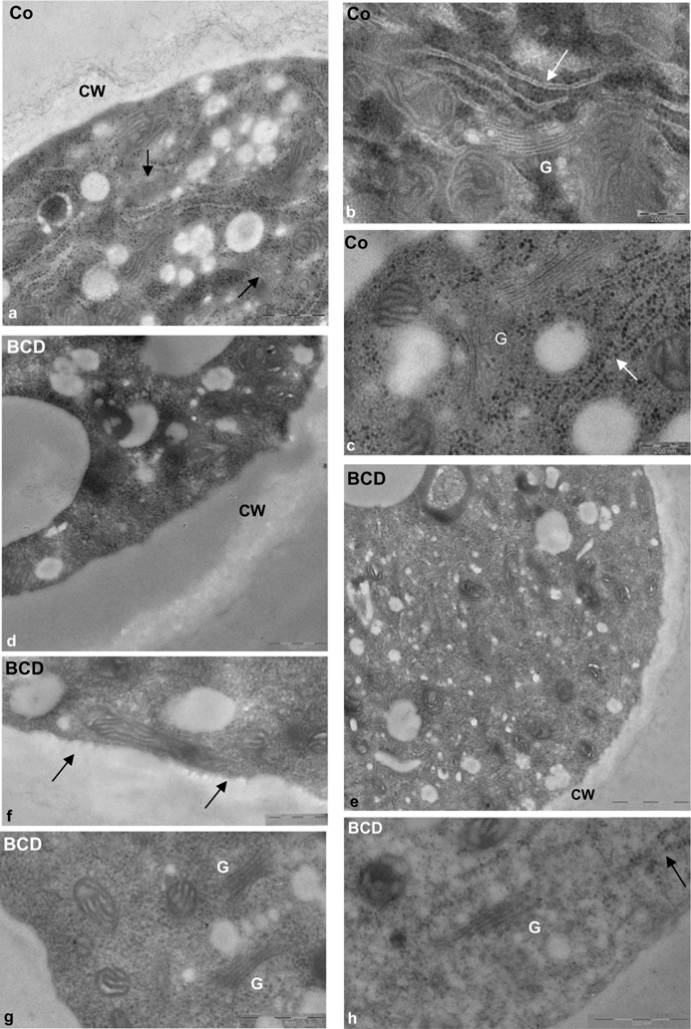
Effect of BCD on pollen tube morphology. (a–c) The cytoplasm of control pollen tubes was rich organelles such as mitochondria, Golgi bodies (a, arrow; b,c, indicated as G) and smooth (b, arrow) and rough ER (c, arrows). The cell wall (a, indicated as CW) showed fibrillar and amorphous components. (d–h) BCD-treated pollen tubes showed a strong reduction in cell wall fibrillar component (d,e). Alterations were observed in PM ultrastructure (f, arrows). Golgi bodies (g) and ER elements (h, arrow) were rarely observed in BCD-treated pollen tubes. Scale bars: 200 nm (b,c); 500 nm (a,d; f-h); 1 µm (e).

As BCD induced profound effects on DIM ultrastructure and on the polarised distribution of membrane microdomains, causing slow pollen tube growth, we investigated the effect of BCD on pollen tube cytoplasm morphology. Pollen tubes grown in the presence of 16 mM BCD for 2 h and 30 min were processed for transmission electron microscopy and compared with control cells. Sterol depletion dramatically affected pollen tube morphology. The PM of control pollen tubes was straight (Fig. 11a) whereas that of BCD-treated pollen tubes was wavy (Fig. 11e,f, arrows). Changes were also observed in the quantity of membranous organelles. In 15 control pollen tube sections about 90 Golgi bodies were observed (Fig. 11a, arrows; Fig. 11b,c), whereas in the same number of BCD-treated cell sections only three Golgi bodies were found (Fig. 11g,h). Both rough and smooth ER appeared strongly reduced in BCD samples (Fig. 11h, arrow) with respect to control (Fig. 11b,c, arrows). Finally, alterations in cell wall ultrastructure were also observed. In control pollen tubes the cell wall showed both fibrillar and amorphous phases (Fig. 11a), whereas in the cell wall of BCD-treated pollen tubes fibrillar components seemed to be absent (Fig. 11d–h).

## Discussion

Pollen tube growth requires fine integration of signal transduction, cytoskeletal organisation and membrane trafficking to allow polarised secretion (Hepler et al., 2001; Cheung and Wu, 2008; Onelli and Moscatelli, 2013). Although a number of actors involved in these processes have been identified (Ischebeck et al., 2010), the structural constraints to compose them into a clear tableau are still elusive. Examples of these actors are Rho- and Rab-GTPases (Cheung et al., 2002; Kost et al., 1999; Helling et al., 2006; de Graaf et al., 2005), cytoskeleton interacting proteins and enzymes regulating the polarised distribution of lipids (Ischebeck et al., 2011). To have a first view of putative membrane micro-domains in pollen tubes, we both isolated DIMs from pollen tube microsomes and visualised some heterogeneity of pollen tube PM by imaging. The results of the present study add new potential players to the game by revealing the presence of membrane micro-domains in pollen tubes.

It has however been extensively questioned whether DIMs represent membrane micro-domains *in vivo* and whether the characterised DIM proteins are actually associated with such domains *in vivo* (Tanner et al., 2011; Malinsky et al., 2013). As underlined by these authors, the DIM approach followed by proteomic analysis of DIM fractions detects hundreds of proteins which may not necessarily be confirmed as proteins of micro-domains *in vivo*. In addition, specific associations of proteins can be disrupted by the use of detergents, so that protein components that could be of interest could be discarded and missed. We are aware of these intrinsic limitations of the DIM approach but used it as a starting point to screen proteins of potential interest for further research in pollen tube organisation and function. Once a protein has been identified in the DIM fraction, additional approaches such as high resolution imaging (photon or electron microscopy) can be used to confirm its localisation in micro-domains *in vivo*. In animal as in plant cells, sterol depletion experiments with BCD have clearly shown that a good correlation between the presence of proteins in DIMs and their association with membrane micro-domains *in vivo* can be established (Kierszniowska et al., 2009; Pike, 2003; Roche et al., 2008; Raffaele et al., 2009). We are therefore convinced that our first approach has produced enough candidates for further study towards unraveling pollen tube functions.

Although the requirement of polarised distribution of sterols and membrane microdomains has been demonstrated for other tip-growing cells as root hairs (Ovecka et al., 2010) and gymnosperm pollen tubes (Liu et al., 2009), respectively, this aspect had not previously been investigated in Angiosperm pollen tubes.

In growing tobacco pollen tubes two different experimental approaches showed a polarised distribution of sterols and higher order membranes from tip to shank. Live cell imaging also showed that this polarised distribution of high ordered membrane domains in the tip was abolished when cells were incubated with BCD. In addition, the use of BCD also clearly caused disorganisation of isolated DIMs and changes in protein repartitioning between soluble and insoluble membrane fractions and in subcellular distribution of DIM proteins such as VDACs. Finally, BCD also dramatically altered cell wall and PM ultrastructure and secretory system organisation, since both ER and Golgi apparatus were rarely observed in BCD-treated cells, leading to slower pollen tube growth rates. These changes were similar to those observed in somatic plant cells after membrane microdomain disorganisation (Melser et al., 2010; Melser et al., 2011).

Protein analysis showed features of pollen tube DIMs, such as actin, prohibitin and proteins involved in methylation and phosphoinositide regulation. In addition, VDACs, tubulins and proteins involved in membrane trafficking make DIMs likely platforms linking some of the crucial processes involved in pollen tube growth. Altogether, these results sustain the concept that membrane microdomains are involved in Angiosperm pollen tube growth and that isolated DIMs may provide proteins of interest.

### Sterol and sphingolipid-enriched membrane domains

To have a broad view of putative membrane microdomains, we therefore isolated DIMs from pollen tube microsomes. Increasing detergent/protein ratios isolated low density membranes that meet the diagnostic requirements for membrane rafts. Lipid analysis revealed that sterols were the major lipid species in pollen tube DIMs, their content being about three times that of P2, in line with sterol-enrichment reported for DIMs in animal cells (Pike, 2003). This data also suggests that phytosterols could be the major determinants of lipid partition in pollen tube membranes, as shown in experiments *in vitro* (Ahmed et al., 1997; Schroeder et al., 1998; Brown and London, 2000; London and Brown, 2000).

Concerning sphingolipids, the enrichment of glucosylceramide seemed to be modest compared to that of sterols, but may vary according to plant tissue (Cacas et al., 2012). Careful analysis of 2-hydroxylated VLCFAs more typical of the plant sphingolipids glycosyl inositol phosphoryl ceramides and DIMs (Buré et al., 2014; Cacas et al., 2012), than non-hydroxylated VLCFAs, which are less rich in rafts domains, showed that pollen tube DIMs also respond to sphingolipid enrichment. Furthermore, enrichment of phospholipids with saturated acyl chains together with hydroxylated VLCFA (22–26 carbon atoms) of sphingolipids could reinforce the lipid-lipid interface in membrane raft microdomains. Although sphingolipid-enrichment of plant DIMs did not appear as great as that of sterols, sphingolipid depletion has negative effects on plant development. *Arabidopsis* plants deficient in serine palmitoyltransferase, the first enzyme of sphingolipid biosynthesis, showed reduced growth (Chen et al., 2006). Sphingolipid inhibitors affected the secretory pathway and induced structural changes in endomembranes of plant somatic cells (Melser et al., 2010; Markham et al., 2011). Because pollen tube DIMs were isolated from microsomes, they were more heterogeneous than those obtained from somatic cell PM, observed by TEM (Peskan et al., 2000; Mongrand et al., 2004; Lefebvre et al., 2007). In particular, DIMs isolated from tobacco leaf PM showed rounded vesicles with diameters of 100–400 nm and thin tubular projections (Peskan et al., 2000), similar to those observed in pollen tube DIMs. Observation of DIMs after chemical fixation and sectioning revealed ribbon-like membranes, like those of DIMs isolated from tobacco and *Medicago truncatula* PM. Exposure of pollen tubes to BCD induced disorganisation of DIMs, sustaining the presence of lipid rafts domains.

### Membrane domain role in coupling tip-localised signalling cascade to cytoskeleton

Protein analysis of pollen tube DIMs revealed proteins that were not identified in DIMs isolated from somatic cell PM (Mongrand et al., 2004; Borner et al., 2005; Lefebvre et al., 2007). Although this could reflect the peculiarity of tip- versus isodiametric-growing cells, it cannot be excluded that these proteins are derived from endomembrane DIMs. Our experiments showed that post-translationally modified forms of actin likewise partition between L_o_ and L_d_ domains of pollen tube microsomes. The current raft model asserts that raft-associated proteins, like actin, contribute to the formation of L_o_ nanodomains and to their clustering into large platforms (Douglass and Vale, 2005; Chichili and Rodgers, 2009). In animal cells there is a strong link between membrane rafts and cortical actin filaments (AFs) because the fraction of ordered domains decreases or increases according to whether AFs depolymerise or stabilise (Dinic et al., 2013). In pollen tubes, cortical AFs are localised in the shank where they establish physical connections with the PM (Lancelle and Hepler, 1991), while a dynamic actin fringe is believed to deliver SVs to the apical flanks (Zonia and Munnik, 2008; Bove et al., 2008; Moscatelli et al., 2012). Although we can hypothesise that insoluble actin associates with raft domains in the shank and apical PM, the nature of this interaction is not known. Intriguingly, pollen tubes stained with filipin showed a periodic concentration of sterols in the shank PM, reminiscent of the ring-like deposition of pectins and GPI-anchored AGP proteins in lily pollen tubes (Li et al., 1994; Li et al., 1996). This observation suggests that raft microdomains in the shank could have a periodic distribution and that insoluble actin could mediate the interaction between cortical AFs and the PM and/or influence PM organisation. Other possibilities are that insoluble actin is a storage form of the protein or that it functions for formin-dependent AF nucleation. In fact, an emerging feature of formins (FH) is their association with membranes, especially with the PM, either as transmembrane proteins or via the phosphoinositide phosphatase PTEN domains (Cvrčková, 2013). AFH1 and AFH3 formins were shown to nucleate AFs axially distributed on the PM in *Arabidopsis* pollen tubes (Cheung and Wu, 2004; Ye et al., 2009), whereas AFH5 associates with the apical flanks of the pollen tube PM, where it directly stimulates actin nucleation (Cheung et al., 2010). It is known that PIP_2_, which links the PM to actin dynamics, is enriched in animal and plant DIMs (Parmryd et al., 2003; Furt et al., 2010) .In the pollen tube apex, the localisation of AFH5 coincides with that of RhoGTPase and PIP_2_ (Cheung et al., 2010; Fu et al., 2001), both members of the signalling pathway regulating Ca^2+^ homeostasis and actin polymerisation (Kost et al., 1999; Vincent et al., 2005; Ischebeck et al., 2011), and intriguingly also overlaps with the sterol-enriched and higher membrane-order PM platform that delimits the tip. This evidence, together with the presence of GPI-anchored COBRA-like 10 protein (Li et al., 2013), makes this domain a good candidate for the role of platform integrating RhoGTAPase/PIP_2_ production with actin polymerisation and targeted secretion. AF depolymerisation experiments using DIM proteins as markers could help to reveal the mutual relationships between rafts and cortical AFs in shank and apex.

Interestingly, SAC6, a suppressor-of-actin (SAC) domain phosphatase (Hughes et al., 2000), is also present in pollen tube DIMs. In plant cells, modulation of the phosphoinositide pool plays important roles in regulating signal transduction, AF organisation and membrane trafficking in polarised plant cells (de Graaf et al., 2005; Preuss et al., 2006). The *Arabidopsis* genome contains nine SAC domain proteins with different phosphoinositide substrates, but only SAC6 is specifically expressed in pollen (Zhong and Ye, 2003). Studies on SAC1 showed that it localises in Golgi apparatus, while the AtSAC1 gene in the *fragile fiber7* (*fra7*) mutant determines defects in fibre and vessel cell wall thickness and affects actin organisation but not MTs in elongating cells (Zhong et al., 2005). In particular, SAC1p-like phosphoinositide phosphatase RHD4 colocalises with the marker for polarised membrane trafficking, RabA4b, in the tip vesicles of wild type root hairs. Analysis of *Atrhd4-1* revealed that changes in the phosphoinositide pattern alter the localisation of RabA4b and affect tip growth (Thole et al., 2008). It would be interesting to investigate where SAC6 localises in pollen tubes and how changes in phosphoinositide homeostasis affect membrane trafficking or influence the level and localisation of PIP_2_ in the tip PM, consequently affecting actin and raft membrane domain organisation.

Besides insoluble actin, about 30% of membrane bound tubulin was also recovered in DIMs. Studies on animal cells showed that association of tubulin with the PM is mediated by MT-associated proteins (Laezza et al., 1997; Oda et al., 1995) or may occur by a direct insertion of palmitoylated tubulin into the PM (Zambito and Wolff, 1997; Zambito and Wolff, 2001). In plants, membrane-associated tubulin, partly solubilised by non-ionic detergents, was demonstrated in cauliflower cells but its role was not understood (Sonesson et al., 1997). In somatic cells, recent studies showed that cortical microtubules (MTs) are directly involved in positioning transmembrane cellulose synthase (CESA) complexes in specific PM domains (Crowell et al., 2009; Gutierrez et al., 2009), thus regulating cellulose microfibril orientation and cell morphogenesis (Li et al., 2012; Lei et al., 2014). In addition to interaction with cortical MTs, the lipid environment also seems to play a role in the correct organisation and functioning of CESA complexes, and they, in turn, could contribute to PM organisation (Guerriero et al., 2008; Schrick et al., 2012). This data suggests that together with cortical MTs, CESA interacts with specific lipids to form membrane microdomains, thus explaining the presence of tubulins in DIMs isolated from plant cell PM (Mongrand et al., 2004; Borner et al., 2005; Lefebvre et al., 2007). In pollen tubes, MTs form an extensive cortical network running parallel to AFs in the shank (Idilli et al., 2013; Lancelle and Hepler, 1992) and are organised in short, randomly oriented segments in the apex (Idilli et al., 2013). Interestingly, CESA complexes were recently localised on clear zone vesicles and in very tip PM, whereas crystalline cellulose is observed 5–15 µm behind the tip (Ferguson et al., 1998), suggesting that cellulose is deposited in the apex in a disorganised way and is further organised and oriented parallel to cortical MT bundles in the apical flanks (Guerriero et al., 2008; Guerriero et al., 2014). The disorganisation of higher ordered membrane by BCD, in the tip region and reduction of the fibrillar component of the cell wall suggests that the proper positioning and functioning of CESA complexes is linked to the integrity of membrane microdomains in pollen tubes.

Conversely, it remains to be determined how insoluble tubulin binds to DIMs. More experiments are needed to understand the relationship between sterol-enriched domains and CESA complexes and in general the link between DIMs and MTs in pollen tubes.

### Membrane microdomains and membrane trafficking

MALDI-TOF/MS analysis revealed proteins involved in membrane trafficking. The γ-2 subunit of the coatomer complex, regulating the sorting of GPI-proteins into ceramide-enriched COPII vesicles in their route from ER to the *cis*-face of Golgi apparatus (Muñiz et al., 2001; Guillas et al., 2003), was identified in pollen tube DIMs. The γ-2 subunit is also part of the COPI coatomer that mediates intra-Golgi and Golgi-to-ER vesicle trafficking (Beck et al., 2009). In polarised epithelial cells, the sorting of GPI-anchored proteins to the apical surface occurs in sterol-sphingolipid-rich domains/vesicles of the TGN (Simons and Ikonen, 1997). Pollen tube growth requires insertion of Golgi derived SVs in the tip region to provide cell wall material and new tracts of PM. The presence of the γ-2 subunit of the coatomer complex suggested that raft domains and coatomer complexes cooperate in vesicle delivery to sort proteins destined for the apex from those to be secreted in the shank (Silva et al., 2010; Moscatelli et al., 2012; Idilli et al., 2013). Studies on endocytosis also revealed a mechanism of membrane sorting in the tip (Parton et al., 2001; Helling et al., 2006; Moscatelli et al., 2007; Idilli et al., 2013); in particular, the localisation of pollen tube Rab11 homolog, a marker of vesicles involved in secretory/membrane recycling activity in the apex (de Graaf et al., 2005; Szumlanski and Nielsen, 2009; Idilli et al., 2013), partly overlaps with sterol- and membrane ordered domain- enriched vesicles, suggesting that membrane domains could be involved in sorting newly internalised vesicles to vacuoles or in recycling proteins/lipids to different PM domains of the apex (Helling et al., 2006; Idilli et al., 2013). As a matter of fact, BCD affected the subcellular localisation of VDACs, leading the VDACs being mislocalised to vacuoles and ER, suggesting that membrane microdomains participate in membrane trafficking.

Interestingly, data from di-4-ANEPPDHQ live cell imaging also showed that BCD induced changes in membrane lipid organisation level in clear zone vesicles, so it could be interesting to investigate whether sterol depletion affects RabA4d localisation and membrane sorting in the apex.

Pollen tube growth also requires efficient membrane recycling (Onelli and Moscatelli, 2013). In particular, clathrin-dependent and -independent endocytosis have been observed to convey materials to vacuoles in the apex and shank of the tube and to recycle PM to Golgi apparatus, respectively (Moscatelli et al., 2007; Zhao et al., 2010; Idilli et al., 2013). Proteins involved in PM internalisation and endosome trafficking, such as a clathrin heavy chain isoform 5, the clathrin interacting protein epsin and two dynamin-like proteins, were found in pollen tube DIMs. During clathrin-dependent endocytosis, dynamins are required for invagination and release of clathrin-coated vesicles (Conner and Schmid, 2003). Dynamin 1C colocalises with clathrin-coated vesicles in the cell plate of dividing cells, where it is presumably involved in recycling excess secreted PM (Otegui et al., 2001; Seguí-Simarro et al., 2004), whereas in pollen tubes it is present in the apex and in the subapical PM, where clathrin is also concentrated (Blackbourn and Jackson, 1996; Moscatelli et al., 2007; Konopka et al., 2008). Since raft domains can be involved in clathrin-independent endocytosis (Kirkham and Parton, 2005; Eyster et al., 2009), the presence of clathrin and dynamins in DIMs is controversial. However, cells treated with the sterol inhibitor fenpropimorph showed altered dynamics of DRP1C and clathrin light chain on the PM (Konopka et al., 2008), suggesting that the lipid environment plays a role in the efficiency of clathrin-dependent endocytosis. On the other hand, clathrins and dynamins are also involved in membrane trafficking from the TGN to vacuoles (Sawa et al., 2005) and since both clathrin and dynamins showed a cytoplasmic localisation, it could not be excluded that clathrin and dynamins cooperate with membrane rafts to promote vesicle budding during membrane trafficking (Konopka et al., 2008; Moscatelli et al., 2007).

It is argued that membrane microdomains, modulating the composition and function of endocytic and secretory systems, also regulate pollen tube PM structure. In pollen tubes, the accumulation of sterols and higher order membrane in the apical PM and in clear zone vesicles suggest that membrane microdomains play a role in cell polarisation. According to observations in *Picea meyeri* pollen tubes (Liu et al., 2009), sterol depletion also destroyed the localisation of ordered membrane domains in tobacco pollen tube PM, suggesting a conservation mechanism maintaining PM asymmetry in the male gametophyte through plant evolution.

Studies on sterol-deficient *Arabidopsis* mutants and sterol depletion experiments revealed radical changes in cell wall ultrastructure as well as defects in Golgi organisation and DIM delivery to the PM in somatic cells (Schrick et al., 2004; Laloi et al., 2007; Melser et al., 2011). Analogously, sterol depletion also disorganises the secretory system and profoundly alters the PM ultrastructure in pollen tubes.

### Proteins involved in methylation reactions

Protein analysis of pollen tube DIMs also revealed the presence of the zinc-binding methyl transferase, S-adenosylmethionine synthase (MetE). This enzyme catalyses *de novo* synthesis of methionine (Matthews and Goulding, 1997) and is part of the S-methylmethionine (SAM) cycle that can also enter several other metabolic pathways (Ranocha et al., 2000). In fact, MetE also regenerates the methyl group of SAM, a cofactor required as methyl donor in a large number of methylation reactions (Ranocha et al., 2001). MetE was identified in tobacco pollen tubes where it was localised on vesicles partly distributed in cytoplasm and that accumulate in the clear zone (Moscatelli et al., 2005), where sterol-rich vesicles and ordered membrane microdomains are also localised.

Intriguingly, a methyltransferase PMT3-like protein was also discovered in pollen tube DIMs, suggesting that MetE and PMT3 cooperate in methylation reactions in the sterol biosynthetic pathway (Nes et al., 2003) to regulate the sterol patterns and sorting activity of transport vesicles in the clear zone.

### Mitochondria-plastid proteins or stress-responsive proteins

Mitochondrial and plastid proteins have been discovered in pollen tube DIMs as well as in DIMs isolated from plant somatic cells (Mongrand et al., 2004; Borner et al., 2005; Lefebvre et al., 2007).

Three different forms of voltage dependent anion channels (VDACs, spots 8, 10, 11) and one porin (spot 11) are enriched in pollen tube DIMs with respect to L_o_ membrane domains. VDACs were first identified as a major component of the mitochondrial outer membrane in eucaryotes and observed to form pores for the diffusion of small solutes, as well as contributing to energy production and apoptosis (Benz, 1994; Godbole et al., 2003). In addition, VDAC1 is associated with caveolae, PM regions rich in sterols and sphingolipids in animal cells (Lisanti et al., 1994; Schindler et al., 2006), while two alternative splice variants of mouse VDAC1 were found to be directed to the secretory pathway or to mitochondria (Buettner et al., 2000).

In plants, VDACs are involved in innate immunity by regulation of H_2_O_2_ production (Tateda et al., 2009; Tateda et al., 2011). Analysis of *Arabidopsis* VDACs revealed that dicotyledons have two classes of this protein, one presenting the mitochondrial porin sequence (MPS) at the C-terminus and the other showing a divergent MPS motif (Tateda et al., 2009), and that three of the four VDACs expressed in *Arabidopsis* are differentially associated with mitochondria and PM (Robert et al., 2012). Accordingly, immunolocalisation of VDACs in *Lotus japonicus* and soybean root nodules demonstrated their presence not only on mitochondria but also on cell cortex vesicles (Wandrey et al., 2004). One main question concerning the presence of VDACs in pollen tube DIMs is whether they are derived from mitochondria or from cell membranes. Sequence analysis of the three VDACs identified in pollen tube DIMs showed that all of them have the MPS motif (supplementary material Fig. S4B, red sequence), suggesting the presence of raft domains in pollen tube mitochondria.

As DIMs were prepared from microsomal fractions, mitochondrial membranes may have contaminated DIMs. Western blot analysis of pollen tube DIMs using an antibody against a mitochondrial marker polypeptide confirmed that mitochondrial DIMs were present in our preparation. Immunolocalisation experiments using a polyclonal antibody against VDAC also revealed that, VDACs are localised on cytoplasmic vesicles other than on the mitochondrial outer membrane in pollen tubes, analogously to other cell types (Wandrey et al., 2004), suggesting that DIMs containing VDACs are partly derived from cytoplasmic vesicles. However, the recruitment of raft domains in mitochondria has been reported in animal cells in response to stress stimuli (Garofalo et al., 2005; Ciarlo et al., 2010), and the possible presence of membrane microdomains in pollen tube mitochondria could also be investigated.

Pollen tube DIMs also displayed the stress-response protein, prohibitin. In yeast and mammals, the two prohibitin subunits (PHB1 and PHB2) associate to form a chaperone complex in the inner mitochondrial membrane, playing a role in folding of newly synthesised proteins (Nijtmans et al., 2000) and also localising in the nucleus to control the cell cycle (Wang et al., 2002). In plants, PHBs are involved in maintaining mitochondrial efficiency and exert a positive effect on plant development, cell proliferation and senescence (Ahn et al., 2006; Van Aken et al., 2007; Chen et al., 2005; Jiang et al., 2006). In addition, since PHB synthesis increases after biotic and abiotic stress in different species of Angiosperms, it was postulated that PHBs are also involved in preserving mitochondrial function by regulating ROS levels (Ahn et al., 2006; Van Aken et al., 2009; Wang et al., 2010). Tip growth also implies tip localised production of ROS by NADPH oxidase (Potocký et al., 2007; Liu et al., 2009; Wang et al., 2010) that integrates with the RhoGTPase/Ca^2+^ signalling cascade (Potocký et al., 2012). Moreover, it has been shown that sterol depletion alters ROS production and dissipates the tip-focused Ca^2+^ gradient, leading to pollen tube arrest in *Picea meyeri* pollen tubes (Liu et al., 2009). The presence of PHBs in pollen tube DIMs could be involved in protecting mitochondrial function in response to the physiological production of ROS during pollen tube growth.

## MATERIALS AND METHODS

### Fluorescent probes and drugs

Filipin and di-4- ANEPPDHQ (Invitrogen, USA) were dissolved in DMSO to a concentration of 10 mg/ml and 3 mM and then diluted to final concentration of 50 µM and 1 µM, respectively. β-cyclodextrin (BCD) (Sigma Aldrich, USA) was dissolved directly in the pollen culture medium to final concentration of 8 mM and 16 mM.

### Pollen culture and pollen tube crude extracts

For DIM isolation, electron microscopy and filipin staining *Nicotiana tabacum* (L.) pollen was collected from plants in the Botanical Garden Città Studi of Milan University, dehydrated by incubation for 12 h in a box containing silica gel and then stored at −20°C. Before germination, pollen was hydrated in a humid chamber overnight. Pollen (2.5 mg/ml) was germinated in BK medium (Brewbaker and Kwack, 1963) containing 12% sucrose at 23±2°C.

Pollen tube growth assays in the presence of BCD were carried out in BK medium. Pollen was allowed to germinate for 90 min, then 8 mM and 16 mM BCD were added directly to the culture medium and pollen tubes were left to grow for 2 h and 30 min. Control and treated pollen tubes were fixed as reported previously (Idilli et al., 2013) and observed by inverted microscope (Axiovert 200M, Zeiss) using a 10× objective. Images were collected with a cooled camera (Axiocam HRM Rev. 2). The lengths of control and treated pollen tubes were calculated by ImageJ software (National Institutes of Health) (Schneider et al., 2012) and analysed by (Student's *t*-test) using the program Excel.

For crude extract, pollen tubes were resuspended in two volumes of PEM buffer (100 mM Pipes pH 6.8, 5 mM EGTA, 1 mM MgCl_2_, 1 mM DTT, 1 mM PMSF, 10 µg/ml TAME, 10 µg/ml leupeptin, 10 µg/ml pepstatin A, 4 µM aprotinin, 8 µM antipain) and homogenised on ice in a 2 ml Potter homogeniser. Laemmli sample buffer was added to the homogenate and the sample was boiled for 2 min. It was then centrifuged at 4°C for 36 min at 20,627 ***g*** (15,000 r.p.m.) in an ALC A21-C rotor. The resulting supernatant was collected as crude extract.

### DIM preparation

*Nicotiana tabacum* (L.) pollen was collected and cultured as reported above. Pollen tubes grown for 2 h and 30 min with or without 8 mM and 16 mM (BCD) were rinsed with 20 ml of incomplete TNE buffer (50 mM Tris-HCl pH 7.4, 150 mM NaCl, 5 mM EDTA, 10 µl/ml TAME, 1 mM PMSF) with or without 8 mM and 16 mM BCD, containing 12% sucrose and centrifuged at 2000 r.p.m. for 10 min at 10°C in a Beckmann JS13.1 rotor. Pollen tubes were homogenised on ice in two volumes of complete TNE buffer (50 mM Tris-HCl pH 7.4, 150 mM NaCl, 5 mM EDTA, 1 mM PMSF, 10 µg/ml TAME, 10 µg/ml leupeptin, 10 µg/ml pepstatin A, 4 µM aprotinin, 8 µM antipain) using a 5 ml Potter (teflon/glass) homogeniser. The microsomal fraction (P2) was prepared following Moscatelli et al., 2007. The P2 pellet was resuspended in cold TNE buffer and ice cold 10% (w/v) Triton X 100 was added to a 2:1 to 12:1 detergent/P2 protein mixture (1% final concentration). When the effect of BCD was tested on DIMs, the same amount of P2 protein from control and BCD-treated pollen tubes was incubated with Triton X-100. After treatment with 1% Triton for 30 min on ice, membranes were mixed with 60% Optiprep (from Sigma) to a final concentration of 40% (v/v), placed at the bottom of a centrifuge tube and overlaid with successive 3 ml steps of 35%, 30% and 15% (v/v) Optiprep/TNE buffer. Gradients were centrifuged for 19 h at 169,000 ***g*** (37,000 r.p.m.) in a Beckmann SW41 Ti rotor. DIMs were recovered as an opaque band of floating material at the interface between 15% and 30%. Gradients were partitioned into 19 fractions and aliquots of each fraction were denatured for electrophoresis. Fractions corresponding to the floating material were pooled, diluted with 4 vol of TE buffer (50 mM Tris-HCl pH 7.4, 5 mM EDTA, 1 mM PMSF, 10 µg/ml TAME) and centrifuged at 105,000 ***g*** (32,000 r.p.m.) for 2 h at 4°C in a Beckmann SW 60 Ti rotor. The pellet, resuspended with TE buffer, was assayed for protein concentration by the Bradford protein assay (Bradford, 1976). Aliquots of P2 and DIMs were dried in a Speed Vac SC110 (Savant) and stored at −80°C for further analysis of lipid profile and polypeptide composition.

### HPTLC analysis of lipids

Lipids were extracted with chloroform/methanol (2:1, v/v) for 30 min at room temperature and then washed with 0.9% NaCl. The solvent was evaporated and lipid extracts were dissolved in an appropriate volume of chloroform/methanol (1:1, v/v). Phospholipids were analysed by loading total lipids onto HPTLC plates (60F254, Merck, Darmstadt, Germany), which were developed in methyl acetate/n-propanol/chloroform/methanol/0.25% aqueous KCl (25:25:25:10:9, v/v) according to Heape et al., (Heape et al., 1985). To isolate and quantify glucosylceramide (Glucer), the lipid extracts were further analysed on HPTLC plates developed with chloroform/methanol (85:15, v/v) (Hillig et al., 2003). To isolate and quantify sterols, total lipids were loaded onto HPTLC plates developed with hexane/ethylether/acetic acid (90:15:2, v/v) as described previously (Laloi et al., 2007). Lipids were identified by co-migration with known standards and quantified by densitometry analysis (Macala et al., 1983) using a TLC scanner 3 (CAMAG, Muttenz, Switzerland) as described previously (Laloi et al., 2007).

### GC-FID and GC-MS analysis of fatty acids

Fatty acid methyl esters were prepared by treating lipid extracts with 1 ml of 2.5% H_2_SO_4_ (v/v) in methanol using heptadecanoic acid 1 µg/ml as internal standard. Tubes were heated at 80°C for 1 h and cooled to room temperature. Then 400 µl hexane and 1.5 ml H_2_O were added to extract fatty acid methyl esters. The tubes were shaken vigorously and centrifuged at 3000 r.p.m. and the organic phases were transferred to injection vials.

GC was performed using a Hewlett-Packard 5890 Series II gas chromatograph equipped with an HP-1 column (30 m × 0.32 mm × 0.25 mm) and a flame ionisation detector (GC-FID) or an Agilent 6850 gas chromatograph equipped with an HP-5MS column (30 m × 0.25 mm × 0.25 mm) and an Agilent 5975 mass spectrometric detector (70 eV, mass-to-charge ratio 50–750) for GC-MS. The same GC program was used in both cases with helium as carrier gas.

### One- and two-dimensional electrophoresis

Proteins were resolved in denaturing 10% acrylamide gels in a discontinuous buffer system (Laemmli, 1970). MiniVe Vertical Electrophoresis System (GE Healthcare, USA) was used for analytical one-dimensional electrophoresis. For preparatory one-dimensional electrophoresis, polypeptides (30 µg DIMs at detergent/protein ratio 8:1 in each lane) were separated using 17 cm × 20 cm, 1.5 mm thick gels (Elettrofor, Rovigo, Italy). Proteins were visualised with Coomassie brilliant blue R250.

For 2D gels, P2 and DIM proteins (30 µg for 2D electrophoresis) were dried by SpeedVac system, dissolved in 2D rehydration buffer, supplemented with 2% 3–10 IPG buffer and loaded onto 7-cm non-linear pH 3–10 strips by overnight passive rehydration at room temperature. IEF was performed with Multifor II system (GE Healthcare, USA) at 200 V for 1 h, 2000 V for 3 h and 3000 V for 3 h and 30 min. Focused strips were equilibrated in Buffer I (0.5 M Tris-HCl, pH 6.8, 2% SDS, 6 M urea, 30% glycerol, 2% DTE) for 12 min and then for another 5 min in Buffer II (composition the same as Buffer I, but with 2.5% iodoacetamide instead of DTE) at room temperature. SDS-PAGE was run in 10% polyacrylamide gel (MiniVe Vertical Electrophoresis System, GE Healthcare, USA), as described previously (Laemmli, 1970). The gels were silver stained (Sinha et al., 2001).

### Western blot and QuantityOne analysis

For western blot, polypeptides were electrotransferred for 2 h at 300 mA to a polyvinylidene difluoride transfer membrane (GE Healthcare, USA), on ice as described previously (Towbin et al., 1979). Membranes were blocked in 5% non-fat milk in Tris-buffered saline (TBS) at room temperature. They were probed with primary monoclonal antibodies diluted 1:2000 overnight at room temperature (B-5-1-2 and 16-B6, for actin and tubulin, respectively; purchased from Sigma Aldrich, USA). Polyclonal antibodies anti-glutamine oxoglutarate aminotransferase (GOGAT) and anti-cytochrome oxidase subunit II (COX II) (from Agrisera, Sweden) were used 1:1000 final concentration. The polyclonal antibody against the voltage dependent anion-selective channel protein 1 (VDAC1) (from Agrisera, Sweden) was used 1:5000 final dilution. Bound antibodies were detected with enhanced chemiluminescence reagent ECL (Thermo Scientific). All gels and western blot images were scanned using Epson Perfection V750 PRO and Adobe Photoshop software. Western blotting data was quantified using the volume analysis tool of QuantityOne 4.6.3 1D Analysis Software (Bio-Rad, Hercules, CA, USA), with Adjusted Volume (Intensity*mm2) as quantitative parameter, and the values expressed as means ± standard errors from two independent sets of data.

### PDQuest analysis

Gels were scanned using Epson Perfection V750 PRO and Adobe Photoshop software. 2D gel profiles were screened for differentially expressed proteins by PDQuest Advanced 8.0.1 2D Gel Analysis Software (version 7.0, BioRad, Hercules, CA, USA Bio-Rad). The images were cropped to the same size and shape and spots were detected and matched automatically to a master gel selected by the software. Spot detection and matching were edited manually. The analysis comprised spot detection, gel matching and statistical analysis. The separate analysis of each sample included alignment of each DIM gel to its reference image (P2). Quantitative analyses were carried out after normalizing spot volumes in all gels to compensate for abundance-related variations. Selection of differentially expressed protein spots was based on fold change abundance >2.0, with a consistent change in the replicate gels of the two biological replicates.

### Mass spectrometry and protein identification

Protein identification was performed as previously described (Hellman et al., 1995; Soskic et al., 1999). Bands and spots of interest were manually excised, destained in ammonium bicarbonate 2.5 mM and acetonitrile 50% (v/v), and acetonitrile dehydrated. Before protein digestion, 1D gel-resolved proteins were reduced with 10 mM DTE in 25 mM ammonium bicarbonate (1 h at 56°C) and then alkylated with 55 mM iodoacetamide in 25 mM ammonium bicarbonate at room temperature (45 min, in darkness). After an incubation with 50 mM ammonium bicarbonate (10 min), protein bands were acetonitrile dehydrated. 1D and 2D gel-resolved proteins were rehydrated in trypsin solution (Sigma Aldrich, Italy) and in-gel protein digestion was performed by an overnight incubation at 37°C. For MALDI-TOF MS, 0.75 µl of each protein digest was directly spotted onto the MALDI target and air-dried. 0.75 µl of an alpha-cyano-4-hydroxycynnamic acid matrix solution was added to the dried samples and allowed to dry again. Mass spectra were acquired using an Ultraflex III MALDI-TOF/TOF mass spectrometer (Bruker Daltonics, Billerica, MA, United States). Spectra were analysed by Flex Analysis software v.3.0. Peptide mass fingerprinting (PMF) database searching was carried out in NCBInr or Swiss-Prot/TrEMBL databases set for *Viridiplantae* (Green Plants) using Mascot (Matrix Science Ltd., London, UK, http://www.matrixscience.com) on-line-available software with the following settings: experimental and theoretical PMF patterns with a Δmass less than 100 ppm, trypsin as the digestion enzyme, one allowed missed cleavage, carbamidomethylation of cysteine as fixed modification, oxidation of methionine as variable modification. The parameters used to accept identifications were: number of matched peptides, extent of sequence coverage, and probabilistic score (Tables 1 and 2).

Peptide digests not unambiguously identified were further analysed performing peptide sequencing by tandem mass spectrometry. MS/MS analysis was performed on the Ultraflex III MALDI-TOF/TOF instrument and on a nanoscale LC-ESI-MS/MS system using a Micro-HPLC Pump Phoenix 40 (Thermo Finnigan, San Jose, CA) and a LCQ DECA IT mass spectrometer (Thermo) (Bianchi et al., 2013). By the on-line-available MASCOT MS/MS ion search software, MS/MS database searching was carried out in Swiss-Prot/TrEMBL database applying the following parameters: trypsin specificity, one missed cleavage allowed, peptide precursor mass tolerance: 100 ppm, fragment mass tolerance: 1.2 Da, peptide precursor charge state: +2, carbamidomethylation of cysteine as fixed modification, oxidation of methionine as possible modification, and taxonomy: *Viridiplantae* (Green Plants).

For LC-ESI-MS/MS analysis, peptides were extracted in 50% (v/v) acetonitrile and 0.1% (v/v) TFA and concentrated by Speed-Vac (SC110A Savant Speed-Vac, Thermo), then MS/MS peptide sequencing was performed as previously described (Meiring et al., 2002; Bianchi et al., 2013). MS/MS database searching was carried out in Swiss-Prot/TrEMBL database using Mascot MS/MS ion search software. The taxonomy was limited to *Viridiplantae* (Green Plants), peptide precursor charge was set to +2 or +3, and precursor and fragment peptides' mass tolerances were ±1.2 Da and ±0.6 Da, respectively. Trypsin was selected as the digestion enzyme with one allowed missed cleavage. Carbamidomethylation of cysteine was assumed as fixed modification, while oxidation of methionine as possible one. Only peptides with individual ion scores p< 0.05 were significant.

### Transmission electron microscopy

Freshly prepared microsomes and DIMs were laid on copper grids for 30 min and then rinsed twice in 5 mM EGTA for 2 min. Samples were stained in 1% uranyl acetate for 10 sec and air dried before observation. Samples of microsomes and DIMs floating on the Optiprep density gradient were fixed overnight at 4°C, as described previously (Moscatelli et al., 2007). Fixed membranes were resuspended in an equal volume of 5% sodium alginate in 50 mM Hepes pH 7.4 and small drops of suspension were solidified in 0.2 M CaCl_2_ in 50 mM Hepes. Drops were rinsed in Hepes buffer at 4°C for 1 h and post-fixed with 1% osmium tetroxide for 1 h. Samples were dehydrated with increasing concentrations of methanol and embedded in LR GOLD resin according to the manufacturer's protocols (London Resin, London England). Infiltration and polymerisation were done at −20°C with a CS-Auto (Reichert Jung, London England) cryo-substitution apparatus. 80 nm ultra-thin sections were obtained by Ultracut E microtome (Reichert Jung) and collected on nickel grids. Sections were then stained with 3% uranyl acetate for 20 min. Grids were observed with an EFTEM LEO 912AB transmission electron microscope (Zeiss, Jena, Germany) operating at 80 kV.

For BCD experiments pollen was hydrated overnight and then allowed to germinate for about 90 min (2.5 mg/ml) in BK medium before adding 8 mM and 16 mM of BCD for 2 h and 30 min (Sigma Aldrich, Italy). In control assays pollen tubes were cultured in BK medium. For immune-electron microscopy, treated and control cells were collected and processed for EM observation as described previously (Moscatelli et al., 2007). In order to analyse pollen tube ultrastructure in greater detail, control and treated pollen tubes were post-fixed with 1% OsO_4_ in Hepes buffer for 1 h, before dehydration and embedding procedures. Pollen tube sectioning was performed as reported above.

For immunogold labelling, sections of cells that were not post-fixed with 1% OsO_4_ were incubated with 1% bovine serum albumin for 30 min. The specimens were incubated with primary antibody (1:500) for 1 h and rinsed two times with TBS. The 10 nm gold-conjugated secondary antibody (1:400) was incubated for 1 h. Sections were post fixed with 0.5% glutaraldehyde for 15 min and rinsed again with TBS. Control experiments were also performed omitting the primary antibody. Grids were stained with 3% uranyl acetate for 20 min and observed as reported above. The procedure for immunogold labelling was carried out at room temperature.

### Filipin labelling and di-4-ANEPPDHQ ratiometric live imaging

Pollen tubes were cultured and fixed, as described previously (Moscatelli et al., 2012). Samples were mounted using cytifluor (Agar Scientific, UK), TBS and Filipin 50 µM final dilution (Invitrogen, USA, 10 mg/ml stock in DMSO) (Muller et al., 1984). Filipin was excited with the 351- and 362-UV laser lines and the fluorescence emitted was collected between 400 and 490 nm. Cells were imaged with a 63× oil immersion (NA 1.4) objective (Leica Microsystems, GmbH, Wetzlar, Germany). All images were recorded using a stepper motor to make Z-series. Optical sections (0.5 µm) and three-dimensional projections of specimens were obtained by the Leica TCS SP2 confocal microscope.

For di-4-ANEPPDHQ staining of live cells, one drop of pollen tube suspension was mixed with an equal amount of 1% low melting agarose (Sigma Aldrich, USA), dissolved in culture medium, and stratified on polylysinated coverslips (2 mg/ml polylysine for 2 h). Then pollen tubes were overlayered with the medium. The viability of cells was assessed by recording pollen tube growth rate before adding the dye. Cells were loaded with the dye by direct addition of 1 µM di-4-ANEPPDHQ (Invitrogen, USA) for 1 min. Di-4-ANEPPDHQ was imaged with a Leica TCS SP2 confocal microscope. In sterol depletion experiments, pollen tubes were incubated with 16 mM BCD for 2 h before loading the dye.

Confocal images of pollen tubes (single medial sections) were recorded with LCS software sequential scan mode using a 63× oil immersion (NA 1.4) objective and a 2.0 zoom lens (Leica Microsystems, GmbH, Wetzlar, Germany). Ratiometric, pseudo-colored general polarisation images were obtained from di-4-ANEPPDHQ fluorescence by ImageJ (National Institutes of Health) (Schneider et al., 2012), using the method developed by Owen et al. (Owen et al., 2012). Disordered (620–750 nm) and ordered (500–580 nm) channels were used to obtain the image ratio, setting an intensity threshold of 15 for both. The mean red/green ratio values in the tip (white ROI) and shank (yellow ROI) regions were calculated by ImageJ, excluding pixels with a value of 0 (background pixels). Statistical analysis was performed by Student's *t*-test.

## Supplementary Material

Supplementary Material
