# Supplementary material for: Characterisation of detergent-insoluble membranes in pollen tubes of Nicotiana tabacum (L.)
Source: Biol Open. 2015 Feb 20;4(3):378–99. doi: 10.1242/bio.201410249 (PMC4359744; doi:10.1242/bio.201410249)
Supplement: Supplementary Material [file supp_4_3_378__index.html]

Characterisation of detergent-insoluble membranes in pollen tubes of Nicotiana tabacum (L.) — Characterisation of detergent-insoluble membranes in pollen tubes of Nicotiana tabacum (L.) — Supplementary Material 

# Characterisation of detergent-insoluble membranes in pollen tubes of *Nicotiana tabacum* (L.)

## bio.201410249 Supplementary Material

**Files in this Data Supplement:**

- Supplementary Material - Alessandra Moscatelli et al. doi: 10.1242/bio.201410249
