# Supplementary material for: Characterisation of detergent-insoluble membranes in pollen tubes of Nicotiana tabacum (L.)
Source: Biol Open. 2015 Feb 20;4(3):378–99. doi: 10.1242/bio.201410249 (PMC4359744; doi:10.1242/bio.201410249)
Supplement: Supplementary Material [file supp_bio.201410249_bio.201410249-s1.pdf]

Biology Open (2015) 000, 1–22 doi:10.1242/bio.201410249

Supplementary Material

Alessandra Moscatelli et al. doi: 10.1242/bio.201410249

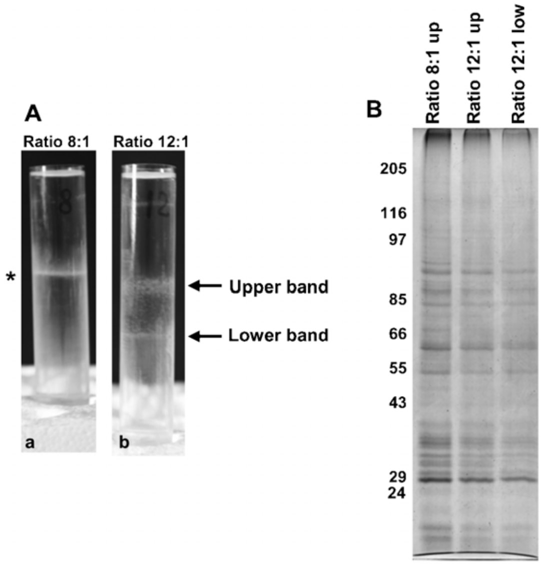

Fig. S1. DIM purification. (A) Optiprep gradients showing the single floating band in ratio 8:1 (*) and the two floating bands in ratio 12:1. (B) Electrophoretic profile of DIMs obtained using detergent/protein ratios of 8:1 and 12:1, respectively; upper and lower band in ratio 12:1 showed similar electrophoretic pattern.

Biology Open (2015) 000, 1–22 doi:10.1242/bio.201410249

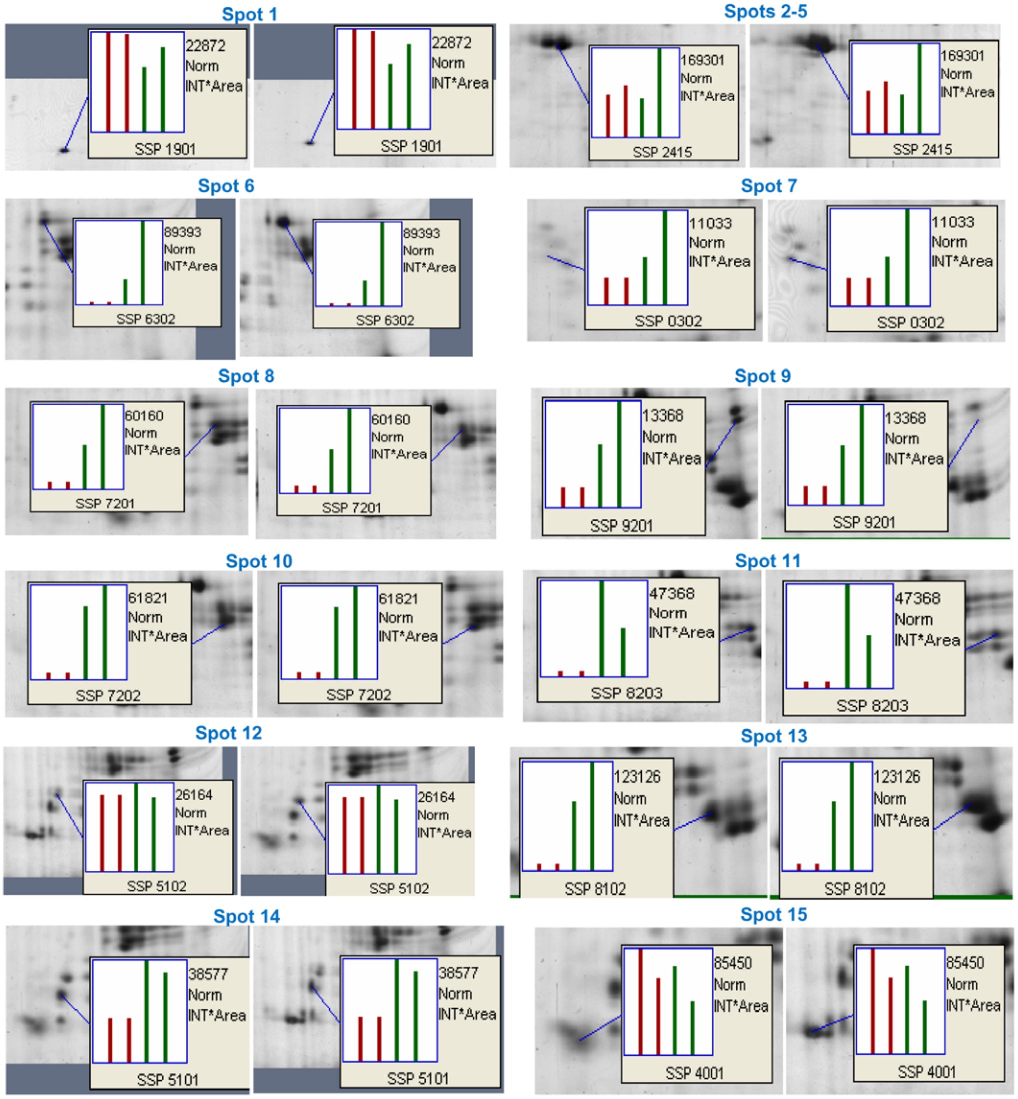

Fig. S2. PDQuest analysis of 2D gels, showing the amount of identified DIM polypeptides, with respect to the corresponding P2 polypeptides.

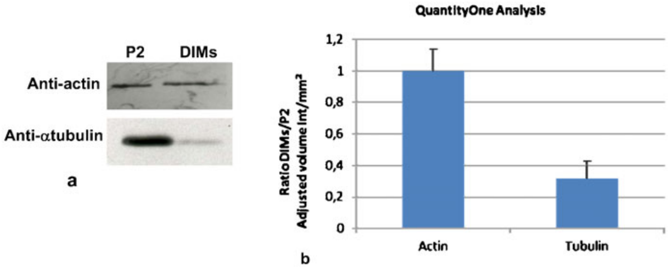

Fig. S3. QuantityOne analysis. (a) Anti-actin and anti-α tubulin western blotting in DIMs and P2. (b) Graph showing the amount of actin and tubulin recovered in DIMs with respect to P2, respectively (n=3). Error bars indicate standard errors.

Biology Open (2015) 000, 1–22 doi:10.1242/bio.201410249

A
Actins

```
gi|6103623      MADAEDIQPLVCDNGTGMVKAGFAGDDAPRAVFPSIVGRPRHTGVMVGMGQKDAYVGDEA 60
ACT_GOSHI       MADGEDIQPLVCDNGTGMVKAGFAGDDAPRAVFPSIVGRPRHTGVMVGMGQKDAYVGDEA 60
gi|224112777    MAEAEDIQPLVCDNGTGMVKAGFAGDDAPRAVFPSIVGRPRHTGVMVGMGQKDAYVGDEA 60
gi|224088196    MAESEDIQPLVCDNGTGMVKAGFAGDDAPRAVFPSIVGRPRHTGVMVGMGQKDAYVGDEA 60
ACT1_TOBAC      MADGEDIQPLVCDNGTGMVKAGFAGDDAPRAVFPSIVGRPRHTGVMVGMGQKDAYVGDEA 60
                **:.********************************************************

gi|6103623      QSKRGILTLKYPIEHGIVSNWDDMEKIWHHTFYNELRVAPEEHPVLLTEAPLNPKANREK 120
ACT_GOSHI       QSKRGILTLKYPIEHGIVNNWDDMEKIWHHTFYNELRVAPEEHPVLLTEAPLNPKANREK 120
gi|224112777    QSKRGILTLKYPIEHGIVSNWDDMEKIWHHTFYNELRVAPEEHPVLLTEAPLNPKANREK 120
gi|224088196    QSKRGILTLKYPIEHGIVSNWDDMEKIWHHTFYNELRVAPEEHPVLLTEAPLNPKANREK 120
ACT1_TOBAC      QSKRGILTLKYPIEHGIVSNWDDMEKIWHHTFYNELRVAPEEHPVLLTEAPLNPKANREK 120
                ******************.*****************************************

gi|6103623      MTQIMFETFNVPAMYVAIQAVLSLYASGRTTGIVLDSGDGVSHTVPIYEGYALPHAILRL 180
ACT_GOSHI       MTQIMFETFNAPAMYVAIQAVLSLYASGRTTGIVLDSGDGVSHTVPIYEGYALPHAILRL 180
gi|224112777    MTQIMFETFNTPAMYVAIQAVLSLYASGRTTGIVLDSGDGVSHTVPIYEGYALPHAILRL 180
gi|224088196    MTQIMFETFNTPAMYVAIQAVLSLYASGRTTGIVLDSGDGVSHTVPIYEGYALPHAILRL 180
ACT1_TOBAC      MTQIMFETFNTPAMYVAIQAVLSLYASGRTTGIVLDSGDGVSHTVPIYEGYALPHAILRL 180
                **********.*************************************************

gi|6103623      DLAGRDLTDALMKILTERGYSFTTTAEREIVRDVKEKLAYVALDYEQELETAKSSSSIDK 240
ACT_GOSHI       DLAGRDLTDALMKILTERGYSFTTTAEREIVRDVKEKLAYIALDYEQELETSKTSSSIEK 240
gi|224112777    DLAGRDLTDSLMKILTERGYSFTTTAEREIVRDMKEKLAYIALDYEQELETSKTSSTVEK 240
gi|224088196    DLAGRDLTDALMKILTERGYSFTTTAEREIVRDMKEKLAYIALDYEQELETAKTSSSVEK 240
ACT1_TOBAC      DLAGRDLTDHLMKILTERGYSFTTTAEREIVRDVKEKLSYIALDFEQEMETSKTSSSVEK 240
                ********* ***********************:****:*:***:***:**:*:**:::*

gi|6103623      SYELPDGQVITIGAERFRCPEVLFQPSLIGMEAAGIHETTYNSIMKCDVDIRKDLYGNIV 300
ACT_GOSHI       SYELPDGQVITIGAERFRCPEVLFQPSMIGMEAAGIHETTYNSIMKCDVDIRKDLYGNIV 300
gi|224112777    SYELPDGQVITIGAERFRCPEVLFQPSMIGMEAAGIHETTYNSIMKCDVDIRKDLYGNIV 300
gi|224088196    SYELPDGQVITIGAERFRCPEVLFQPSMIGMEAAGIHETTYNSIMKCDVDIRKDLYGNIV 300
ACT1_TOBAC      SYELPDGQVITIGAERFRCPEVLFQPSMIGMEAAGIHETTYNSIMKCDVDIRKDLYGKIV 300
                ***************************:*****************************:**

gi|6103623      LSGGSTMFPGIADRMSKEITALAPSSMKIKVVAPPERKYSVWIGGSILASLSTFQQMWIA 360
ACT_GOSHI       LSGGTTMFPGIADRMSKEITALAPSSMKIKVVAPPERKYSVWIGGSILASLSTFQQMWIA 360
gi|224112777    LSGGSTMFPGIADRMSKEISALAPSSMKIKVVAPPERKYSVWIGGSILASLSTFQQMWIA 360
gi|224088196    LSGGSTMFPGIADRMSKEITALAPSSMKIKVVAPPERKYSVWIGGSILASLSTFQQMWIA 360
ACT1_TOBAC      LSGGSTMFPGIADRMSKEITALAPSSMKIKVVAPPERKYSVWIGGSILASLSTFQQMWIA 360
                ****:**************:****************************************

gi|6103623      KAEYDESGPSIVHRKCF 377
ACT_GOSHI       KAEYDESGPSIVHRKCF 377
gi|224112777    KAEYDESGPSIVHRKCF 377
gi|224088196    KAEYDESGPSIVHRKCF 377
ACT1_TOBAC      KAEYDESGPSIVHRKCF 377
                *****************
```

B
Voltage Dependent Anion Channels

```
gi|161788874    MVKGPGLYSDIGKKARDLLYRDYVSDHKFTVTTYSSTGVAITSSGLKKGELFLADVNTQL 60
VDAC2_SOLTU     MVKGPGLYSDIGKKARDLLYRDYVSDHKFTVTTYSTTGVAITASGLKKGELFLADVSTQL 60
gi|161788872    MGKGPGLYSDIGKRARDLLYRDYQTDHKFTITTYSPTGVAITSSGLKKGDLFLADVNTQL 60
gi|161788876    MGKGPGLYTEIGKKTRDLLYKDYQSDHKFTITTYSPTGVAITSSGTKKGDLFLADVNTQL 60
                * ******::***::*****:** :*****:****.******:** ***:******.***

gi|161788874    KNKNVTTDVKVDTNSNVYTTITVDEPAPGLKTIVSFVLPDQRSGKVELQYLHEYAGISTG 120
VDAC2_SOLTU     KNKNITTDVKVDTNSNVYTTITVDEPAPGLKTIFSFVVPDQKSGKVELQYLHEYAGINTS 120
gi|161788872    KNKNVTTDIKVDTNSNLLATVTVDEAAPGLKTILSFRIPDQSSAKLEVQYLHDYAGISTS 120
gi|161788876    KNKNITTDIKVDTNSNLFTTITVDDAAPGLKTILSFRVPDQRSGKLEVQYLHEYAGISTS 120
                ****:***:*******: :*:***:.*******.** :*** *.*:*:****:****.*.

gi|161788874    IGLTASPLVNFSGVAGNNTVALGTDLSFDTASGNFTKCNAGLSFSTSDLIASLALNDKGD 180
VDAC2_SOLTU     IGLTASPLVNFSGVAGNNTVALGTDLSFDTATGNFTKCNAGLSFSSSDLIASLALNDKGD 180
gi|161788872    LGLTANPIVNFSGVVGTNLLSLGTDLSFDTKVGAITKCNAGLSFTNTDLIASLNLNDKGD 180
gi|161788876    VGLTANPIVNFSGVVGTNILALGTDVSFDSKTGDFTKCNAGLSFTNADLVASLNLNDKGD 180
                :****.*:******.*.* ::****:***:  * :*********:.:**:*** ******

gi|161788874    TLSASYYHTVKPVTNTAVGAELTHSFSSNENTLTIGTQHLLDPLTTVKARVNSYGKASAL 240
VDAC2_SOLTU     TVSASYYHTVKPVTNTAVGAELTHSFSSNENTLTIGTQHLLDPLTTVKARVNSYGKASAL 240
gi|161788872    ALSASYYHIVSPLTMTAVSAEVTHSFCTNENTISVGTQHQLDPLTTVKARVNNLGKANAL 240
gi|161788876    NLSASYYHSVSPFTSTAVGAEVNHCFSTNENTITVGTQHRLDPLTSVKARVNNFGKASAL 240
                 :****** *.*.* ***.**:.*.*.:****::**** *****:******. ***.**

gi|161788874    IQHEWRPKSLFTISGEVDTRAIEKSAKIGLAVALKP 276
VDAC2_SOLTU     IQHEWRPKSLFTISGEVDTRAIEKSAKIGLAVALKP 276
gi|161788872    IQHEWRPKSLFTISGEVDTKAVDKSAKFGLALALKP 276
gi|161788876    LQHEWRPKSLFTISGEVDTKSVDKGAKFGLALALKP 276
                :******************::::*.**:***:****
```

C
Prohibitins

```
gi|568815637    MGSNQAAVSFLTN--IARAAFGLGIGATV----LNSSLYTVDGGQRAVLFDRFRGVIDDT 54
gi|71370259     MNLNNVKVPKMPGGGAASALIKLGVVAGLGVYGVANSLYNVDGGHRAIVFNRIIGVKDKV 60
                *. *:. *. :..    * * : **: * :     : .***.****:**::*:*: ** *..

gi|568815637    VGEGTHFLVPWLQKPFIFDIRTRPHTFSSVSGTKDLQMVHLTLRVLSRPEVSRLPYIFKN 114
gi|71370259     YPEGTHFMIPWFERPVIYDVRARPHLVESTSGSRDLQMVKIGLRVLTRPVPDQLPTVYRT 120
                  *****::**:::*.*:*:*:***  ..*.**::*****:: ****:**  .:** :::.

gi|568815637    LGLEYDEKVLPSIGNEVMKAVVAQFNADQLLTERPQVSALVRESLIRRAKDFNIVLDDVA 174
gi|71370259     LGENYNERVLPSIIHETLKAVVAQYNASQLITQRENVSREIRKILTERAANFNIALDDVS 180
                **  :*:*:*****  :*.:******:**.**:*:* :**   :*: * .** :***.****:

gi|568815637    ITHLSYGAEFSKAVEQKQVAQQEAERSKFVVMKAEQERRAAIIRAEGESESAKLISDATA 234
gi|71370259     ITSLTFGKEFTAAIEAKQVAAQEAERAKFVVEKAEQDKRSAIIRAQGEAKSAQLIGQSIA 240
                ** *::* **: *:* **** *****:**** ****::*:*****:**::**:**.:: *

gi|568815637    AAGMGLIELRRIEASREVASTLAKTPNVAYLPKQGNMLLALSPTR------- 279
gi|71370259     NN-PAFITLRKIEAAREIAQTMSHAANKVYLSSD-DLLLNLQDLNLDGAGKR 290
                    .:* **:***:**:*.*::::.* .**..: ::** *.   .
```

Fig. S4. ClustalW analysis.

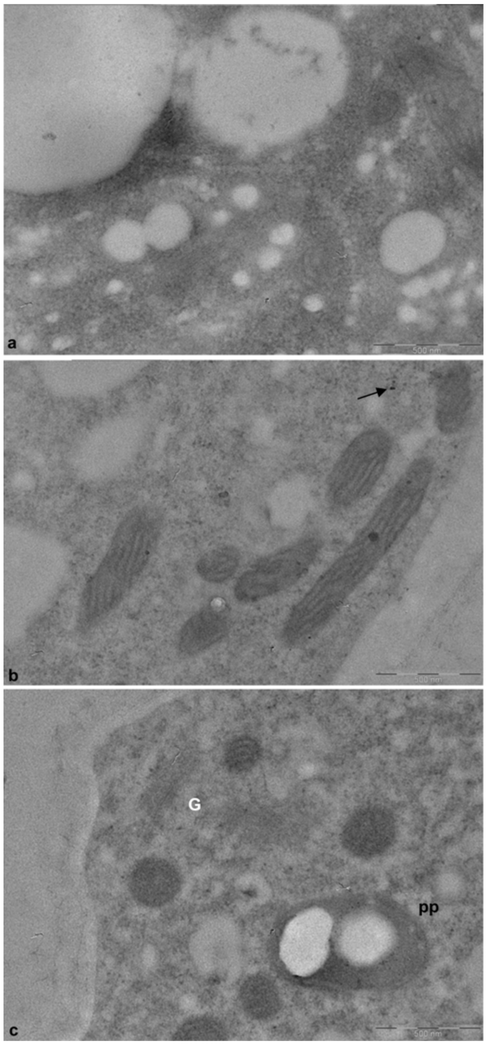

Fig. S5. Immunogold labelling control experiments in which the primary antibody was omitted. (a–c) Any membranous organelle was stained by the secondary antibody (G=Golgi body; pp=proplastids. (b) Rare gold particles were seldom observed in the cytoplasm (arrow). Scale bars: 500 nm (a-c).

Biology Open

Biology Open (2015) 000, 1–22 doi:10.1242/bio.201410249

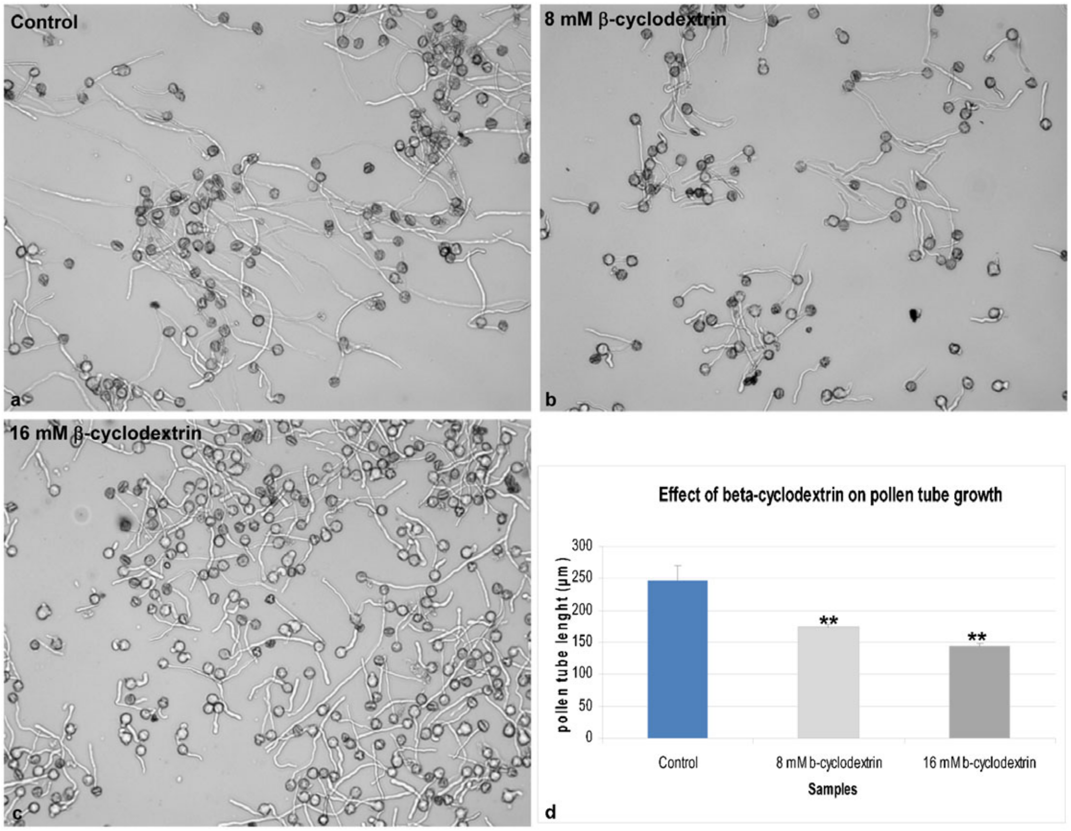

Fig. S6. Inhibition of the pollen tube growth rate by BCD. (a–d) The pollen tube growth was affected by 8 mM and 16 mM BCD, with respect to the control. Error bars represent standard errors. ** indicate $P<0.001$.

Biology Open
